# Topical Recombinant Human Epidermal Growth Factor for Oral Mucositis Induced by Intensive Chemotherapy with Hematopoietic Stem Cell Transplantation: Final Analysis of a Randomized, Double-Blind, Placebo-Controlled, Phase 2 Trial

**DOI:** 10.1371/journal.pone.0168854

**Published:** 2017-01-03

**Authors:** Ji-Won Kim, Myeong Gyu Kim, Hyun Jung Lee, Youngil Koh, Ji-Hyun Kwon, Inho Kim, Seonyang Park, Byoung Kook Kim, Jung Mi Oh, Kyung Im Kim, Sung-Soo Yoon

**Affiliations:** 1 Department of Internal Medicine, Seoul National University Bundang Hospital, Seongnam, Republic of Korea; 2 College of Pharmacy, Seoul National University, Seoul, Republic of Korea; 3 Department of Internal Medicine, Dongguk University Ilsan Medical Center, Goyang, Republic of Korea; 4 Department of Internal Medicine, Seoul National University Hospital, Seoul, Republic of Korea; 5 Cancer Research Institute, Seoul National University, Seoul, Republic of Korea; 6 Biomedical Research Institute, Seoul National University Hospital, Seoul, Republic of Korea; 7 Department of Internal Medicine, Chungbuk National University Hospital, Cheongju, Republic of Korea; 8 Research Institute of Pharmaceutical Sciences, Seoul National University, Seoul, Republic of Korea; 9 College of Pharmacy, Korea University, Sejong, Republic of Korea; 10 Biomedical Research Center, Korea University Guro Hospital, Seoul, Republic of Korea; Tata Memorial Centre, INDIA

## Abstract

The aim of this study was to evaluate the efficacy and safety of recombinant human epidermal growth factor (rhEGF) oral spray for oral mucositis (OM) induced by intensive chemotherapy with hematopoietic stem cell transplantation. In this phase 2 study, patients were randomized to either rhEGF (50 microg/mL) or placebo in a 1:1 ratio. The primary endpoint was incidence of National Cancer Institute (NCI) grade ≥2 OM. A total of 138 patients were enrolled in this study. In the intention-to-treat analysis, rhEGF did not reduce the incidence of NCI grade ≥2 OM (p = 0.717) nor reduce its duration (p = 0.725). Secondary endpoints including the day of onset and duration of NCI grade ≥2 OM, the incidence of NCI grade ≥3 OM and its duration, and patient-reported quality of life were also similar between the two groups. In the per-protocol analysis, however, the duration of opioid analgesic use was shorter in the rhEGF group (p = 0.036), and recipients in the rhEGF group required a lower cumulative dose of opioid analgesics than those in the placebo group (p = 0.046), among patients with NCI grade ≥2 OM. Adverse events were mild and transient. This study found no evidence to suggest that rhEGF oral spray reduces the incidence of OM. However, further studies are needed to investigate the effect of rhEGF on OM-induced pain reduction after intensive chemotherapy.

## Introduction

Chemotherapy-induced oral mucositis (OM), a condition characterized by erythema, edema, and ulceration of the oral cavity, is one of the most common and debilitating adverse effects in patients undergoing intensive chemotherapy and hematopoietic stem cell transplantation (HSCT) [1–3]. Many complications including oral pain, odynodysphagia, dysgeusia, malnutrition, dehydration, and increased risk of systemic infections caused by OM can influence not only a patient’s quality of life (QoL), but also the outcomes of chemotherapy. Although many trials have been targeted towards preventing or treating OM, palifermin (recombinant human keratinocyte growth factor) is currently the only approved drug for the prevention of severe OM in patients with hematological malignancies undergoing autologous HSCT after total body irradiation (TBI) plus high-dose chemotherapy [4].

Epidermal growth factor (EGF) is a single-chain polypeptide composed of 53 amino acids, and exists in a number of tissues and fluids in the body [5, 6]. It can stimulate the proliferation and differentiation of epithelial tissue and facilitate skin regeneration and wound healing [7, 8], suggesting that it might be effective for treatment of chemotherapy- or radiotherapy-induced OM. Indeed, a previous animal study revealed that recombinant human EGF (rhEGF) enhanced the mucosal wound-healing process [7, 9, 10], while topical application of rhEGF also showed promising therapeutic efficacy and minimal toxicity on radiation-induced OM in patients with head and neck cancer [11, 12].

A pre-planned interim analysis of the current study indicated that rhEGF was generally well tolerated, and produced better outcomes than placebo in terms of several secondary endpoints [13]. In this study, we present the final analysis of this trial to evaluate the efficacy and safety of rhEGF oral spray for chemotherapy-induced OM.

## Patients and Methods

### Study design and participants

This study was a phase 2, randomized, placebo-controlled, double-blind, single-center trial conducted at Seoul National University Hospital, Seoul, Republic of Korea. The study design and protocol were described in detail previously [13]. Patients aged 18 years or older with a documented hematological malignancy who were scheduled to receive intensive chemotherapy followed by autologous or allogeneic HSCT were included. Additional inclusion criteria were a normal oral cavity at baseline, defined as grade 0 according to the National Cancer Institute (NCI) Common Terminology Criteria for Adverse Events (CTCAE) version 3.0, and an Eastern Cooperative Oncology Group performance status of 0–2 [14]. Patients were excluded if they had previously received chemotherapy, radiotherapy, or surgery within the previous 3 weeks, or had a history of allergy to the investigational product or other similar drugs. Patients were also required not to have participated in other clinical trials that could potentially affect the results of this study within the previous 4 weeks. The protocol for this trial and supporting CONSORT checklist are available as supporting information; see S1 Checklist, S1 Protocol (Korean), and S2 Protocol (English).

### Ethics

This trial was registered at www.ClinicalTrials.gov as number NCT00845819 before initiation. The study was approved by the institutional review board (IRB) of Seoul National University Hospital, Seoul, Republic of Korea (IRB number: H-0809-001-255). All participants signed written informed consent before study entry. This clinical trial was conducted according to the Declaration of Helsinki.

### Randomization and masking

Patients were randomly assigned to rhEGF (Easyef^®^ topical solution 0.005%; Daewoong Pharmaceutical Company, Seoul, Republic of Korea) or placebo in a 1:1 ratio, using a computer-generated randomization protocol, by the Medical Research Collaborating Center, Seoul National University Hospital. The study was double blinded, and all clinicians, patients, and investigators responsible for assessing outcomes and analyzing data were masked to treatment assignments. The placebo comprised all the ingredients of the rhEGF preparation except for rhEGF, and was supplied to patients in a masked manner.

### Procedures

Either 50 μg/mL rhEGF or placebo as spray was applied to over the entire oral mucosa twice daily from the first day of conditioning chemotherapy until the day of absolute neutrophil count recovered more than 1,000/μL for 3 days and OM had resolved. Patents were instructed to spray the study drug or placebo on the palate, oropharynx, both buccal mucosa, tongue, and gingiva, a total of six times for each application and to swallow any residue remained in the oral cavity. Patients were also instructed not to eat or drink for 30 min after spraying. During the study period, the investigators verified patient-documented administration records and the remaining volume of the spray to check and remind proper application of the study spray in each patient every day.

Researchers graded the severity of OM daily throughout the study period using NCI CTCAE version 3.0, which classifies the severity of OM by its morphology (grade 0 = normal; grade 1 [mild] = erythema of mucosa; grade 2 [moderate] = patchy ulcerations or pseudomembranes; grade 3 [severe] = confluent ulcerations or pseudomembranes; grade 4 [life-threatening] = tissue necrosis, significant spontaneous bleeding) [14]. Information on clinical laboratory values and use of total parenteral nutrition (TPN), opioid analgesics, and antibiotics was also collected daily. Adverse events related to the investigational product or placebo were graded and reported according to NCI CTCAE version 3.0 [14].

Patient-reported QoL was scored daily during the study period using a modified version of the Oral Mucositis Daily Questionnaire (OMDQ) [15], which consists of questions assessing the patient’s overall health (0–10 scale); severity of mouth and throat soreness (MTS, 0–4 scale); difficulties with swallowing, drinking, eating, talking, and sleeping due to MTS (0–4 scale); and overall MTS status (0–10 scale). For the overall health questions, a higher score denoted a more favorable response. In contrast, a higher score for the other questions denoted a worsening of the MTS-related symptoms. For the analysis of modified OMDQ, the area under the curve (AUC) was calculated from day -8 to day +28 using the trapezoidal method [16].

### Outcomes

The primary endpoint of the current study was the incidence of NCI grade ≥2 OM. Secondary endpoints included the day of onset and duration of NCI grade ≥2 OM, the incidence of NCI grade ≥3 OM and its duration, and patient-reported QoL based on the modified OMDQ throughout the study period. Additional secondary endpoints included the incidence and duration of TPN administration; incidence and duration of oral, parenteral, or transdermal opioid analgesic use and their cumulative dose; incidence of febrile neutropenia and blood infections (bacterial or fungal); duration of antimicrobial treatment; and length of hospital stay. The safety endpoints were the incidence and severity of adverse events related to topical rhEGF administration.

### Statistical analysis

A sample size of 62 patients for each group was sufficient for this study to detect a difference of 27% in the incidence of NCI grade ≥2 OM between two groups with 80% power and a two-sided significance level of 5%, based on the results of a previous study [12, 17]. Considering a dropout rate of 10%, a minimum of 69 patients would be required to complete the study for each group.

Interim analysis was planned at the time of study design using the O’Brien-Fleming type alpha spending function by Lan and DeMets [18]. The significance levels for the interim and final analyses were 0.003 and 0.047, respectively. As initially planned, an interim analysis was performed when 50% of patients were enrolled [13]. This analysis showed that the primary endpoint was not reached. Due to this result, the study did not stop early, but continued as planned.

Statistical analyses were initially conducted in accordance with the intention-to-treat principle. Analysis was also performed for those patients who completed the study (per-protocol analysis). Categorical variables were presented as values and percentages, and were compared using Pearson’s chi-square or Fisher’s exact tests. The Fisher's exact test was indicated when the expected frequency in a cell was less than 5. Continuous variables were summarized as median values and ranges. The Mann–Whitney U test was used to compare differences between two independent groups consisting of continuous variables, which were not normally distributed. Multivariable logistic regression analysis was carried out to estimate the effect of the types of intensive chemotherapy on the primary endpoint, adjusting for the effect of perceived imbalance between the treatment groups. Analysis was performed using IBM SPSS Statistics version 22.0 (IBM, Armonk, NY, USA).

## Results

### Patients

**Fig 1:**
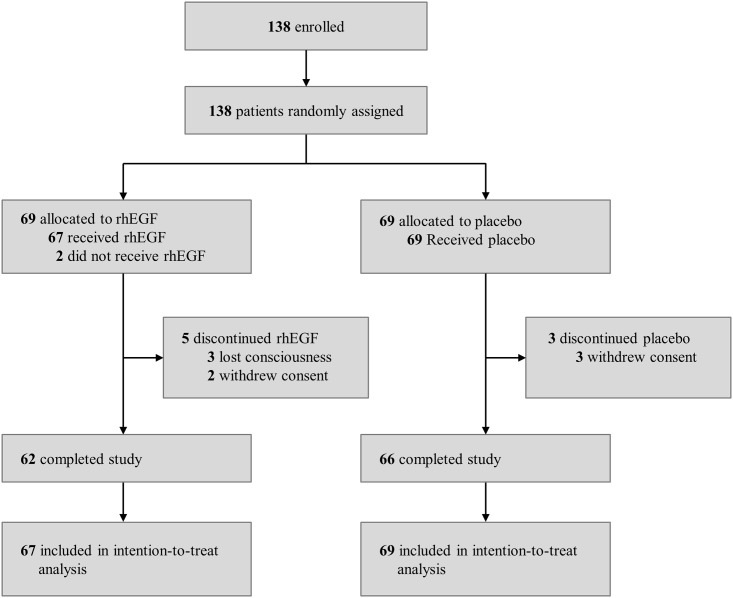
Trial profile.

A total of 138 patients who were scheduled to receive HSCT were enrolled in this study between March 2009 and August 2014 (Fig 1). Two patients withdrew their consent before the study drugs were given. Early treatment discontinuation was the result of loss of consciousness in three patients and withdrawal of consent in five patients.

The baseline demographic and clinical characteristics were similar between the two groups (Table 1). The median age of the 136 patients was 52.0 years (range, 18.0–65.0), and 51.5% of the patients were male. Most patients were diagnosed with multiple myeloma or lymphoma (126 patients, 92.6%) and most underwent autologous transplantation (130 patients, 95.6%). Nearly all the patients received melphalan-containing chemotherapy regimens as conditioning chemotherapy for HSCT, including high-dose melphalan (77 patients, 56.6%), mitoxantrone–etoposide–cytarabine–melphalan (26 patients, 19.1%), or busulfan–etoposide–cytarabine–melphalan (24 patients, 17.6%). The median patient compliance rate for the study drug during the course of treatment was 93.3% (range, 35.0–100.0%) in the rhEGF group and 91.7% (range, 17.7–100.0%) in the placebo group (p = 0.377).

**Table 1:** Patient demographics and baseline characteristics.

Characteristics	rhEGF (n = 67)	Placebo(n = 69)
Age, years	53.0 (18.0–65.0)	51.0 (19.0–65.0)
Gender		
Male	33 (49.3)	37 (53.6)
Female	34 (50.7)	32 (46.4)
Disease type		
Multiple myeloma	38 (56.7)	38 (55.1)
Lymphoma	24 (35.8)	26 (37.7)
Others^a^	5 (4.9)	5 (7.2)
ECOG performance status		
0	38 (56.7)	39 (56.5)
1–2	29 (43.3)	30 (43.5)
HSCT type		
Autologous	64 (95.5)	66 (95.7)
Allogeneic	3 (4.5)	3 (4.3)
Conditioning regimen		
HDM	38 (56.7)	39 (56.5)
NEAM	10 (14.9)	16 (23.2)
BuEAM	13 (19.4)	11 (15.9)
Others^b^	6 (9.0)	3 (4.3)
Chemotherapy intensity		
Dose reduction	13 (19.4)	12 (17.4)
Opioid analgesic use		
Yes	15 (22.4)	14 (20.3)

Data are median (range) or n (%).

Abbreviations: rhEGF = recombinant human epidermal growth factor; ECOG = Eastern Cooperative Oncology Group; HSCT = haematopoietic stem cell transplantation; HDM = high-dose melphalan; NEAM = mitoxantrone–etoposide–cytarabine–melphalan; BuEAM = busulfan–etoposide–cytarabine–melphalan.

^a^Other disease types included acute myeloid leukaemia, acute lymphoblastic leukaemia, amyloidosis, chronic lymphocytic leukaemia, and myelodysplastic syndrome.

^b^Others included fludarabine plus busulfan or melphalan, busulfan plus cyclophosphamide, NEAM with rituximab, and bortezomib containing regimens

### Incidence and duration of OM

In the intention-to-treat analysis, there was no significant difference in the incidence of NCI grade ≥2 OM between the rhEGF and placebo groups (56.7% in the rhEGF group *vs* 53.6% in the placebo group; p = 0.717). Mutivariable analysis indicated that the rhEGF did not significantly change the primary endpoint after adjusting for the types of intensive chemotherapy administered (adjusted odds ratio, 1.211 [95% confidence interval, 0.596–2.459]; Table in S1 Table). Among patients with this degree of mucositis, the median duration of NCI grade ≥2 OM was similar between the two groups (p = 0.725). Similar results were observed for other grades of OM (Table 2). In the per-protocol analysis, rhEGF did not significantly reduce the incidence of NCI grade ≥2 OM (56.5% in the rhEGF group *vs*. 53.0% in the placebo group; p = 0.698) nor the duration of OM (5.0 days [range, 1.0–31.0] in the rhEGF group *vs*. 5.0 days [range, 1.0–36.0] in the placebo group; p = 0.645).

**Table 2:** Effects of rhEGF on the incidence, onset, and duration of OM.

Variable	rhEGF (n = 67)	Placebo (n = 69)	p-value
**OM of NCI grade** ≥**2**			
Incidence	38 (56.7)	37 (53.6)	0.717
In patients with NCI grade ≥2 OM			
Time to onset, days	11.0 (4.0–21.0)	10.0 (5.0–19.0)	0.979
Duration, days	6.5 (1.0–31.0)	5.0 (1.0–36.0)	0.725
**OM of NCI grade** ≥**3**			
Incidence	18 (26.9)	18 (26.1)	0.918
In patients with NCI grade ≥3 OM			
Time to onset, days	11.0 (7.0–20.0)	11.5 (5.0–24.0)	0.542
Duration, days	5.5 (1.0–23.0)	10.0 (1.0–33.0)	0.252

Data are median (range) or n (%).

Abbreviations: rhEGF = recombinant human epidermal growth factor; OM = oral mucositis; NCI = National Cancer Institute

### Impact of rhEGF on other secondary endpoints

In the intention-to-treat analysis, rhEGF tended to be superior to placebo without a statistical significance among patients with NCI grade ≥2 OM, in terms of the median duration of opioid analgesic use for OM-induced pain management (1.5 days; range, 0–19.0) compared with the placebo group (6.0 days; range, 0–48.0) (p = 0.077), as well as in the cumulative dose of opioid analgesics administered (27.8 mg of morphine equivalents [range, 0–1393.5] in the rhEGF group *vs*. 135.0 mg [range, 0–3999.0] in the placebo group; p = 0.086). The per-protocol analysis produced positive results for patients with this degree of mucositis. Patients in the rhEGF group received opioid analgesics for OM-induced pain for a shorter duration than patients in the placebo group (1.0 days [range, 0–19.0] in the rhEGF group *vs*. 6.0 days [range, 0–48.0] in the placebo group; p = 0.036). The cumulative dose of opioid analgesics administered for OM-induced pain management was also lower in the rhEGF group (15.0 mg of morphine equivalents; range, 0–1393.5) compared with the placebo group (135.0 mg of morphine equivalents; range, 0–3999.0) (p = 0.046).

There were no significant differences between the groups in the incidence (15 patients [83.3%] in both the rhEGF and placebo groups; p = 1.000) and median duration of TPN administration (8.5 days [range, 0–25.0] in the rhEGF group *vs*. 10.0 days [range, 0–32.0] in the placebo group; p = 0.308). Thirty-five patients in each group developed febrile neutropenia. The median duration of anti-infective drug use was similar between the two groups (10.5 days [range, 0–101.0] in the rhEGF group *vs*. 11.0 days [range, 0–39.0] in the placebo group; p = 0.199).

The modified OMDQ scores among patients with NCI grade ≥2 OM were similar in both groups. There was a numerical difference in favor of rhEGF compared with placebo for limitations in swallowing and drinking in both the intention-to-treat and per-protocol analyses (Table in S2 Table).

### The rhEGF treatment-related adverse events

Adverse events were similar in both groups (Table 3). The most common adverse event in the rhEGF group was nausea (n = 7, 10.4%). The incidence of other adverse events including oral pain, dry mouth, and taste alteration was low. All the adverse events were mild and transient. No grade 3 or 4 adverse events were noted during the study period.

**Table 3:** Adverse events related to topical administration of study drugs.

Variable	rhEGF (n = 67)	Placebo (n = 69)	p-value
Nausea (grade 1 or 2)	7 (10.4%)	13 (18.8%)	0.167
Pain, oral (grade 1 or 2)	1 (1.5%)	1 (1.4%)	1.000^a^
Dry mouth (grade 1)	0 (0%)	1 (1.4%)	1.000^a^
Taste alteration (grade 1)	2 (3.0%)	1 (1.4%)	0.617^a^

Data are n (%).

^a^, Fisher’s exact test was used.

Abbreviations: rhEGF = recombinant human epidermal growth factor

## Discussion

The final analysis of this randomized phase 2 study found no evidence to suggest that rhEGF oral spray reduced the incidence of OM after intensive chemotherapy followed by HSCT. The effects of rhEGF on secondary endpoints in patients with NCI grade ≥2 OM were also similar to those of placebo, except for a beneficial effect on the duration and cumulative dose of opioid analgesics used to manage OM-induced pain.

The results of this study were not in line with those of a previous study, in which rhEGF reduced the incidence of severe OM in patients undergoing radiotherapy with or without chemotherapy for head and neck cancer [12]. This discrepancy may be at least partly explained by the different conditioning regimens used in the two studies (radiotherapy with or without chemotherapy *vs*. chemotherapy only), given that the conditioning regimen is known to be an independent risk factor for OM [19]. The time course and severity of OM thus varies with the type of chemotherapy or radiotherapy, total dosage, fractionation, and duration of treatment [20]. In our study, the incidence of NCI grade ≥2 OM (55.1% among all patients) was lower than previous studies in patients receiving TBI-based conditioning regimens for HSCT, or in patients undergoing concurrent chemoradiotherapy for head and neck cancer [4, 12]. Another possible explanation for the discrepancy in results is the timing of rhEGF administration. As mentioned in our previous interim analysis, it is possible that rhEGF administered during chemotherapy could negatively stimulate the development of OM, because rhEGF can increase mucosal sensitivity to cancer chemotherapy by accelerating buccal mucosal cell division [13]. Similar results were observed in a recent study evaluating the efficacy of palifermin in patients undergoing autologous HSCT with high-dose melphalan conditioning chemotherapy without TBI [21]. In this study, palifermin did not prevent OM nor reduce the OM-related patient burden, in contrast to results in patients who underwent autologous HSCT after TBI-based conditioning regimen without melphalan [4]. The authors of this study explained that suboptimal timing of the post-dose of palifermin may impair mucosal healing and exaggerate oral toxicity through an exaggerated pharmacological effect of hyperkeratosis [21].

Although it was not the primary endpoint of this study, rhEGF recipients with NCI grade ≥2 OM required a lower dose and shorter duration of opioid analgesic use for the management of OM-induced pain, compared with placebo recipients. In the interim analysis of this study, rhEGF oral spray also significantly reduced the duration and dose of opioid analgesics in patients with NCI grade ≥2 OM [13]. Because OM-related pain is one of the most stressful adverse effects from a patient’s perspective [3], pain control, in addition to treatment of the OM itself, is an important component of cancer patient management. Our results regarding the effect of rhEGF on OM-induced pain may thus have significant implications for the management of patients in this setting. Among patients with NCI grade ≥2 OM, OMDQ analysis showed no significant difference between the two groups. However, rhEGF oral spray tended to reduce limitations in swallowing and drinking, and it is possible that the higher use of opioid analgesics in the placebo group might have masked any potential differences between the two groups.

This study had several limitations. First, the types of intensive chemotherapy were heterogeneous; nonetheless, most primarily contained melphalan. Thus, we adjusted for the types of intensive chemotherapy using the mutivariable logistic regression analysis, and yielded the same result for the primary endpoint. Furthermore, we could not explain the biologic mechanism responsible for rhEGF’s efficacy in reducing the use of opioid analgesics. These results should thus be interpreted as hypothesis-generating findings.

In conclusion, the results of this trial indicated that rhEGF oral spray does not reduce the incidence of NCI grade ≥2 OM in patients receiving intensive chemotherapy with HSCT. However, the observation that rhEGF oral spray might have a favorable effect on OM-induced pain in patients with NCI grade ≥2 OM warrants further investigations.

## Supporting Information

S1 Table Effect of rhEGF on the incidence of NCI grade ≥2 OM after adjusting for the type of conditioning regimen administered. (DOCX)

S2 Table Effects of rhEGF on OMDQ in patient with NCI grade ≥ 2 OM. (DOCX)

S1 CONSORT Checklist CONSORT 2010 checklist. (DOCX)

S1 Protocol Study protocol (Korean version). (DOCX)

S2 Protocol English summary of study protocol. (DOCX)
