# Supplementary material for: Topical Recombinant Human Epidermal Growth Factor for Oral Mucositis Induced by Intensive Chemotherapy with Hematopoietic Stem Cell Transplantation: Final Analysis of a Randomized, Double-Blind, Placebo-Controlled, Phase 2 Trial
Source: PLoS One. 2017 Jan 3;12(1):e0168854. doi: 10.1371/journal.pone.0168854 (PMC5207736; doi:10.1371/journal.pone.0168854)
Supplement: S1 Table — (DOCX) [file pone.0168854.s001.docx]

**S1 Table. Effect of rhEGF on the incidence of NCI grade ≥2 OM after adjusting for the types of intensive chemotherapy**

|  | **Placebo (n=69)** | **rhEGF** **(n=67)** | **p-value^a^** | **Adjusted OR**  **(95% CI)** | **p-value^b^** |
| --- | --- | --- | --- | --- | --- |
| **Incidence of NCI grade ≥2 OM** | 53.6% | 56.7% | 0.717 | 1.211  (0.596-2.459) | 0.596 |

^a^, Unadjusted p-value

^b^, P-value adjusted for the types of intensive chemotherapy

Abbreviations: rhEGF=recombinant human epidermal growth factor; NCI=National Cancer Institute; OM=oral mucositis; OR=odds ratio; CI=confidence interval
