# Supplementary material for: Topical Recombinant Human Epidermal Growth Factor for Oral Mucositis Induced by Intensive Chemotherapy with Hematopoietic Stem Cell Transplantation: Final Analysis of a Randomized, Double-Blind, Placebo-Controlled, Phase 2 Trial
Source: PLoS One. 2017 Jan 3;12(1):e0168854. doi: 10.1371/journal.pone.0168854 (PMC5207736; doi:10.1371/journal.pone.0168854)
Supplement: S2 Table — (DOCX) [file pone.0168854.s002.docx]

**S2 Table. Effects of rhEGF on OMDQ in patient with NCI grade** $\text{≥}$ **2 OM**

| **Variable (AUC)** | **rhEGF** | **Placebo** | **p-value** |
| --- | --- | --- | --- |
| **ITT analysis** | **n=38** | **n=37** |  |
| Q1. Overall health | 220.8 (54.5–308.5) | 212.5 (116.5–353.0) | 0.556 |
| Q2. MTS | 22.3 (0–97.0) | 24.0 (0–96.5) | 0.970 |
| Q3. MTS-related limitations in daily activities | | | |
| Q3a. Swallowing | 14.0 (0–98.0) | 21.0 (0–92.0) | 0.622 |
| Q3b. Drinking | 13.0 (0–99.0) | 21.0 (0–86.0) | 0.528 |
| Q3c. Eating | 23.5 (0–98.0) | 25.0 (0–104.0) | 0.783 |
| Q3d. Talking | 9.5 (0–92.0) | 5.0 (0–97.0) | 0.907 |
| Q3e. Sleeping | 7.0 (0–86.0) | 4.0 (0–72.0) | 0.931 |
| Q4. Overall MTS | 53.3 (0–229.0) | 55.0 (0–222.0) | 0.869 |
| **PP analysis** | **n=35** | **n=35** |  |
| Q1. Overall health | 226.0 (121.5–308.5) | 219.0 (116.5–353.0) | 0.378 |
| Q2. MTS | 21.0 (0–95.0) | 24.0 (0–96.5) | 0.724 |
| Q3. MTS-related limitations in daily activities | | | |
| Q3a. Swallowing | 10.0 (0–75.0) | 19.0 (0–92.0) | 0.374 |
| Q3b. Drinking | 10.0 (0–73.5) | 20.0 (0–86.0) | 0.300 |
| Q3c. Eating | 23.0 (0–90.0) | 22.0 (0–104.0) | 0.568 |
| Q3d. Talking | 7.0 (0–74.0) | 5.0 (0–97.0) | 0.813 |
| Q3e. Sleeping | 6.0 (0–72.0) | 4.0 (0–72.0) | 0.768 |
| Q4. Overall MTS | 41.0 (0–229.0) | 48.0 (0–222.0) | 0.715 |

Data are median (range).

Abbreviations: rhEGF=recombinant human epidermal growth factor; OMDQ=Oral Mucositis Daily Questionnaire; NCI=National Cancer Institute; OM=oral mucositis; AUC=area under the curve; ITT=intention-to-treat; MTS=mouth and throat soreness; PP=per-protocol
