# Supplementary material for: Topical Recombinant Human Epidermal Growth Factor for Oral Mucositis Induced by Intensive Chemotherapy with Hematopoietic Stem Cell Transplantation: Final Analysis of a Randomized, Double-Blind, Placebo-Controlled, Phase 2 Trial
Source: PLoS One. 2017 Jan 3;12(1):e0168854. doi: 10.1371/journal.pone.0168854 (PMC5207736; doi:10.1371/journal.pone.0168854)
Supplement: S1 Protocol — (DOCX) [file pone.0168854.s004.docx]

# **연구계획서**

| **악성혈액질환 환자에서 고용량 항암화학요법에 의해 발생하는 구내염에 대한 재조합 인간상피세포성장인자의**  **무작위 2상 임상 연구**  **Randomized Phase II Study of Recombinant Human Epidermal Growth Factor (rhEGF) on Oral Mucositis Induced by Intensive Chemotherapy for Hematologic Malignancies** |
| --- |

2009.01.19.

서울대학교 의과대학 혈액종양내과

교수 윤 성 수

**Protocol outline**

| 연 구 제 목 | 국문: 악성혈액질환 환자에서 고용량 항암화학요법에 의해 발생하는 구내염에 대한 재조합 인간상피세포성장인자의 무작위 2상 임상 연구  영문: Randomized Phase II Study of Recombinant Human Epidermal Growth Factor (rhEGF) on Oral Mucositis Induced by Intensive Chemotherapy for Hematologic Malignancies |
| --- | --- |
| 연 구 목 적 | 악성혈액질환 환자에서 고용량 항암화학요법에 의해 발생하는 구내염(oral mucositis)에 대한 재조합 인간상피세포성장인자 (recombinant human epidermal growth factor, rhEGF)의 유효성 및 안정성을 평가 |
| 연 구 기 관 | 서울대학교병원 혈액종양내과 |
| 연구책임자 | 소속: 서울대학교 의과대학 내과학교실  전공분야: 혈액종양내과  성명: 윤성수 직명: 교수 (서울대학교병원) |
| 연 구 대 상 | 선정 기준   1. 만 18세 이상의 성인 2. 악성혈액질환이 확진 된 자 3. 고용량 항암화학요법을 예정하고 있는 입원 환자 4. ECOG performance status 0-2인 자 5. 본 연구에 참여하고자 서면 동의한 자   제외 기준   1. 본 약제 또는 이와 비슷한 계열의 약제에 과민성을 갖는 자 2. 항암화학요법 이전에 구강 내 궤양 또는 단순 포진이 있는 자. 즉, 중증도 평가도구- National Cancer Institute Common Terminology Criteria for Adverse Events (NCI CTCAE) v3.0 기준 grade 0 이 아닌 경우 3. 중증의 치과 질환이 있는 자 4. 연구 참여 전 3주 이내에 항암화학요법 또는 방사선 치료를 받은 자 5. 연구 참여 전 3주 이내에 외과적 수술을 받은 자 6. 본 연구에 영향을 줄 수 있는 임상시험에 본 연구 참여 전 4주 이내에 참여하였거나 현재 참여 중인 자 7. 악성혈액질환 외에 연구에 영향을 미칠 만큼의 중증 질환을 동반하고 있는 자 8. 중증 정신질환자 또는 약물 및 알코올 중독 환자 9. 임신 중이거나 수유중인 여성 10. 기타, 연구자에 의해 본 연구에 수행이 곤란하다고 판단되는 자 11. 환자가 연구에 동의하지 않는 경우 |
| 연 구 기 간 | 2009.02.01 – 2010. 01.31 |
| 연 구 방 법 | 고용량 항암화학요법 예정인 성인 악성혈액질환 환자 중 연구에 동의하는 환자를 대상으로 치료기간 동안 재조합 인간상피세포성장인자 (recombinant human epidermal growth factor, rhEGF)를 1일 2회 구강점막 부위가 충분히 덮일 정도로 투여하고 고용량 항암화학요법으로 유도된 구내염의 발생과 중증도 및 이상반응을 평가함으로써 재조합 인간상피세포성장인자 (recombinant human epidermal growth factor, rhEGF)의 유효성 및 안전성을 평가한다. |
| 기대효과 및  예상결과 | 항암치료 과정 중 구내염의 높은 발생률과 심각성에도 불구하고 아직까지 효과적인 예방이나 치료법이 거의 없어서 암환자들은 주로 대증적 요법에 의존하고 있는 상황이다. 구강점막의 상피세포를 포함한 다양한 조직세포의 분화 및 성장을 촉진시킨다고 알려져 있는 재조합 인간상피세포성장인자 (recombinant human epidermal growth factor, rhEGF)는 지금까지의 선행연구 결과들로부터 구내염에 대한 치유효과 및 안전성을 기대할 수 있을 것으로 생각된다. 따라서 본 연구는 고용량 항암화학요법을 시행하는 환자에서 재조합 인간상피세포성장인자의 유효성 및 안전성에 대하여 연구함으로써, 이들 환자의 구내염의 효과적인 예방 및 치료법으로서 재조합 인간상피세포성장인자의 역할을 제시하고자 한다. |

**목 차**

| 1. 연구의 명칭 | ------------------------------------------------------ | 1 |
| --- | --- | --- |
| 1. 연구의 실시기관명 및 주소 | ------------------------------------------------------ | 1 |
| 1. 연구의 책임자, 담당자 및 공동연구자   3.1 연구책임자  3.2 공동연구자 | ------------------------------------------------------ | 1 |
| 1. 연구 배경 및 목적   4.1 연구의 배경  4.2 연구의 목적 | ------------------------------------------------------ | 2 |
| 1. 연구약의 제품명, 제형 등   5.1 시험약  5.2 위약 | ------------------------------------------------------ | 5 |
| 1. 임상시험 대상 환자군   6.1 대상 환자군  6.2 선정 기준  6.3 제외 기준 | ------------------------------------------------------ | 7 |
| 1. 연구기간 | ------------------------------------------------------ | 8 |
| 1. 연구방법   8.1 연구방법 개요  8.2 연구 진행  8.3 목표 피험자의 수 및 산출 근거  8.4 비교군 설정  8.5 이중맹검법  8.6 무작위배정법  8.7 투여량, 투여방법, 투여기간  8.8 병용요법  8.9 피험자교육  8.10 관찰 항목, 검사방법  8.11 안전성  8.12 예측 부작용 및 사용상 주의사항 | ------------------------------------------------------ | 8 |
| 1. 평가변수 및 통계분석   9.1. 평가변수  9.2. 통계분석 | ------------------------------------------------------ | 19 |
| 1. 피험자 보상에 관한 규약 | ------------------------------------------------------ | 22 |
| 1. 피험자의 인권 및 윤리 문제   11.1. 윤리원칙  11.2. 피험자 설명  11.3. 피험자 동의  11.4. 피험자에 대한 안전성의 배려  11.5. 피험자의 정보 보호 | ------------------------------------------------------ | 22 |
| 1. 연구의 기대 성과 | ------------------------------------------------------ | 24 |
| 1. 참고문헌 | ------------------------------------------------------ | 25 |

[별첨 자료]

| 별첨1. 환자와 보호자를 위한 설명서 | --------- | 27 |
| --- | --- | --- |
| 별첨2. 연구 참여 동의서 | --------- | 31 |
| 별첨3. NCI Common Terminology Criteria for Adverse Events v3.0 | --------- | 32 |
| 별첨4. WHO Toxicity Criteria - Oral mucositis scoring system scale | --------- | 33 |
| 별첨5. OMDQ (oral mucositis daily questionnaire) | --------- | 34 |
| 별첨6. ECOG Performance Status | --------- | 35 |
| 별첨7. 임상시험약과 부작용과의 인과관계 | --------- | 36 |
| 별첨8. 중대한 이상반응 보고서 | --------- | 37 |
| 별첨9. 증례기록지 | --------- | 38 |
| 별첨10. 피험자 보상에 대한 규약 | --------- | 49 |

**1. 연구의 명칭**

국문: 악성혈액질환 환자에서 고용량 항암화학요법에 의해 발생하는 구내염에 대한 재조합 인간상피세포성장인자의 무작위 2상 임상 연구

영문: Randomized Phase II Study of Recombinant Human Epidermal Growth Factor (rhEGF) on Oral Mucositis Induced by Intensive Chemotherapy for Hematologic Malignancies

**2. 연구의 실시 기관명 및 주소**

서울대학교병원 혈액종양내과, 서울특별시 종로구 연건동 28번지 서울대학교병원

Division of Hematology and Medical Oncology, Department of Internal Medicine, Seoul National University Hospital, 28 Yongon-dong, Chongno-gu, Seoul, Korea

**3. 연구의 책임자, 담당자 및 공동연구자**

**3.1 연구책임자**

- 윤성수 (서울대학교 의과대학 혈액종양내과 교수)

**3.2 공동연구자**

- 김병국 (서울대학교 의과대학 내과학 교수)
- 박선양 (서울대학교 의과대학 내과학 교수)
- 오정미 (서울대학교 약학대학 임상약학 부교수)
- 김인호 (서울대학교 의과대학 내과학 조교수)
- 김지원 (서울대학교병원 내과 전공의)
- 김경임 (서울대학교 약학대학 임상약학 박사과정)

**4. 연구 배경 및 목적**

**4.1 연구의 배경**

4.1.1. 항암화학요법에 의한 구내염 (oral mucositis)

구내염은 항암화학요법의 가장 흔한 부작용 중 하나로 표준용량 항암제를 투여 받는 암 환자 중 약 10~40%, 고용량 항암제 전처치를 받는 골수이식 시행 환자에서는 약 75~85%가 발생하며 두경부암으로 방사선 조사를 받는 환자에서는 약 85~100%에서 발생한다고 알려져 있다^1~2)^. 일반적으로 항암화학요법 및 방사선 치료로 인한 구내염은 용량-제한적 (dose-limiting)인 특성을 지니며^3)^, 전형적으로 비각질화된 구강 내 표면-목젖을 포함한 연구개, 입천장, 뺨 안쪽과 입술, 혀의 옆과 바닥부분에 주로 발생한다^4)^. 구내염의 주요 증상은 궤양, 발적, 부종, 통증, 미각상실, 구강건조증 등이다^5)^. 항암제 투여로 인한 구내염은 주로 항암치료 시작 후 4~5일부터 시작되며, 백혈구 감소증이 동반되지 않은 일반적인 경우 보통 2~3주 사이에 치유되나 조혈모세포 이식을 받은 환자에서는 그 정도가 심하고 기간이 더 길다^6)^.

이러한 구내염은 암환자가 항암치료 중 가장 고통스럽게 경험하는 부작용 중 하나로 구내염의 통증으로 인한 직접적 고통 외에 삶의 질 감소, 입원기간의 장기화 등 간접적 영향으로 환자의 삶의 질 및 치료 결과에도 중요한 요인으로 작용한다. 실제로 항암화학요법을 받는 환자에서, 구내염 지표 (oral mucositis score)가 1점 상승하는 것은 발열 일수, 감염 위험도, 비경구 영양요법(total parenteral nutrition) 일수, 마약성 진통제의 사용, 총 재원일수 및 100일 내 사망률의 증가와 유의한 연관성을 가짐이 규명되었다^7)^. 따라서 환자뿐 아니라 의료진의 입장에서도 구내염의 치료는 항암치료의 결과 측면에서 매우 중요한 요인이라 할 수 있다.

4.1.2. 현재 항암화학요법 유도 구내염 예방 및 치료의 한계

항암치료 과정 중 구내염의 높은 발생률과 심각성에도 불구하고 아직까지 효과적으로 증명된 예방 및 치료법이 거의 없어서, 암환자들은 주로 대증적 요법에 의존하고 있다^8)^. 구내염으로 인한 통증 감소를 위해서는 lidocaine과 같은 국소 마취제 및 마약/비마약성 진통제가 사용되며 국소적인 윤활제의 적용, 구강세척요법이 기본적인 구강 위생 차원에서 가장 많이 사용되는 대증요법에 해당한다. 이 외에도 allopurinol, benzydamine, sucralfate, silver nitrate, glutamine, chlorhexidine 등을 이용한 구강세척요법이 구내염의 예방과 치료에 긍정적인 영향을 끼친다는 소수의 연구결과들이 있으나 연구 결과의 일관성이 부족하여 권장되고 있지 않다^8)^. 최근에는 전세계적으로 성장인자들의 구내염 치료 및 예방 효과에 대한 연구가 활발히 이루어지고 있으며 keratinocyte growth factor (KGF), transforming growth factor (TGF), fibroblast growth factor (FGF), granulocyte colony-stimulating factor (G-CSF), granulocyte macrophage CSF (GM-CSF) 및 epidermal growth factor (EGF)등이 구내염 치료제로서 연구되고 있다^9)^. 이 중 recombinant human keratinocyte growth factor인 palifermin이 유일하게 2004년 FDA로부터 혈액암 환자의 골수 이식 시 구내염의 예방목적으로 허가를 받은 상태이다.

4.1.3. 상피세포성장인자 (Epidermal growth factor, EGF)

상피세포성장인자는 amino acid polypeptide로 혈소판, 대식세포 및 단핵세포에서 생성되며 상피세포와 섬유아세포 상에 존재하는 상피세포성장인자 수용체와 결합함으로써 구강 점막의 상피세포를 포함한 다양한 조직세포의 분화 및 성장을 촉진시킨다^10~12)^. 상피세포성장인자는 인간의 체액 중 특히 혈액, 침, 눈물 등에 많이 분포하고 있으며, 상처치유 동안 상피세포 외에도 섬유아세포 및 평활근세포의 성장을 촉진함이 알려져 있다^13,14)^. Brown 등은 상피세포성장인자가 돼지의 부분 또는 완전 경화된 피부 상처부위의 상피 재생을 유의하게 촉진시킴을 보고하였으며, 쥐에서도 지속적인 상피세포성장인자의 투여가 피부 상처 부위의 신장강도를 유의하게 증가시킴을 확인하였다^14,15)^. 또한 Nanney 등은 사람을 대상으로 한 연구에서 상피세포성장인자가 상처회복 초기단계에서 상피화를 촉진시킴을 확인하였다^11)^. 한편 최근 다수의 연구들은 항암화학요법 및 방사선요법으로 인한 구내염의 중증도와 타액 내의 상피세포성장인자 농도 감소 간에 유의한 연관성이 있음을 확인하였다^16~20)^. Ino 등은 구내염이 있는 두경부암 환자에서 타액 내 상피세포성장인자의 농도가 현저히 감소되어 있음을 보고한바 있다^13)^. 또한 Epstein 등은 방사선 치료를 받는 두경부암 환자에서 방사선에 의해 야기된 구내염이 타액의 양 및 상피세포성장인자 농도와 관련 있음을 관찰하였으며, 타액의 양과 상피세포성장인자의 분비량이 방사선 치료 첫 주에 유의하게 감소하여 치료기간 동안 낮은 상태로 유지됨을 관찰하고 타액 내 상피세포성장인자의 농도가 높을수록 방사선으로 인한 점막손상의 심각도가 낮을 수 있음을 제시하였다^20)^. 이러한 선행연구 결과로부터 본 연구의 시험약인 재조합 인간상피세포성장인자 (recombinant human epidermal growth factor, rhEGF) 외용액제는 구내염의 주요 관련인자로 생각되는 구강 상피세포 및 점막하층의 섬유모세포의 재생 및 증식을 촉진하여 구내염의 치료효과를 나타낼 것으로 기대할 수 있다. 실제로 방사선치료로 인해 구강 내 점막 및 피부손상이 발생한 두경부암 환자를 대상으로 한 재조합 인간상피세포 성장인자 외용액제 임상연구는 방사선에 의한 점막염과 피부염의 회복에 재조합 인간상피세포 성장인자 외용액의 국소도포가 효과적일 수 있는 가능성을 확인하였으며^21,22)^, 현재는 이를 바탕으로 대규모 임상연구가 진행 중에 있다^23)^.

**4.2 연구의 목적**

4.2.1. 일차적 목적 (Primary Endpoint)

- National Cancer Institute Common Terminology Criteria for Adverse Events (NCI CTCAE) v3.0 [별첨3] 기준 grade 2 이상의 구내염이 발생하지 않은 피험자 분률에 양군간 유의한 차이가 있는지를 평가하고자 함

4.2.2. 이차적 목적 (Secondary Endpoint)

- NCI CTCAE v3.0 기준 grade 2 이상의 구내염의 발생기간에 양군간 유의 차이 여부 평가
- 고용량 항암화학요법 시작 후로부터 NCI CTCAE v3.0 기준 grade 2 이상의 구내염 발생까지의 양군간 유의한 차이 여부 평가
- NCI CTCAE v3.0 기준 grade 3 이상의 구내염 발생률 및 지속기간의 양군간 유의한 차이 여부 평가
- WHO Toxicity Criteria - Oral mucositis scoring system scale [별첨4] 기준 grade 2 이상의 구내염 발생률 및 구내염 지속기간의 양군간 유의한 차이 여부 평가
- WHO oral mucositis scoring system scale 기준 grade 3 이상의 구내염 발생률 및 구내염 지속기간의 양군간 유의한 차이 여부 평가
- WHO oral mucositis scoring system scale 기준 grade 4 이상의 구내염의 발생률 및 지속기간의 양군간 유의한 차이 여부 평가
- Oral Mucositis Daily Questionnaire (OMDQ, [별첨5]) 점수의 양군간 유의한 차이 여부 평가
- 구내염으로 인한 마약성 진통제 총 투여 일수의 양군간 유의한 차이 여부 평가
- 재조합 인간상피세포 성장인자의 부작용 및 안전성 평가

**5. 연구약의 제품명, 제형 등**

**5.1. 시험약**

5.1.1. 성분명

- 재조합 인간상피세포성장인자 (recombinant human epidermal growth factor, rhEGF)

5.1.2. 제품명

- 이지에프외용액 0.005%

5.1.3. 원료의약품 분량

- 1병(10mL) 중,

| 주성분 | 재조합 인간 상피세포 성장인자 (숙주: Escherichia coli JM101, 벡터: pTE105) | 별규 | 0.5mg |
| --- | --- | --- | --- |
| 완충제 | 인산일수소나트륨 | 약전 | 3.58mg |
| 등장화제 | 염화나트륨 | 약전 | 8.77mg |
| pH 조정제 | 인산 | 엔.에프 | 적량 |
| 용제 | 정제수 | 약전 | 적량 |

- 용제부 9mL 중,

| 점증제 | 폴록사머 407 | 엔.에프 | 600mg |
| --- | --- | --- | --- |
| 보존제 | 파라옥시벤조산 메틸 | 약전 | 20mg |
| 완충제 | 인산일수소나트륨 | 약전 | 30.44mg |
| 등장화제 | 자일리톨 | 약전 | 250mg |
| pH 조정제 | 인산 | 엔.에프 | 적량 |
| 용제 | 정제수 | 약전 | 적량 |

5.1.4. 저장방법

- 기밀용기, 냉장보관 (2~8℃)

**5.2. 위약**

5.2.1. 제품명

- 이지에프외용액 0.005% (임상용 위약)

5.2.2. 원료의약품 분량

- 1병(10mL) 중,

| 주성분 | (없음) |  |  |
| --- | --- | --- | --- |
| 완충제 | 인산일수소나트륨 | 약전 | 3.58mg |
| 등장화제 | 염화나트륨 | 약전 | 8.77mg |
| pH 조정제 | 인산 | 엔.에프 | 적량 |
| 용제 | 정제수 | 약전 | 적량 |

- 용제부 9mL 중,

| 점증제 | 폴록사머 407 | 엔.에프 | 600mg |
| --- | --- | --- | --- |
| 보존제 | 파라옥시벤조산 메틸 | 약전 | 20mg |
| 완충제 | 인산일수소나트륨 | 약전 | 30.44mg |
| 등장화제 | 자일리톨 | 약전 | 250mg |
| pH 조정제 | 인산 | 엔.에프 | 적량 |
| 용제 | 정제수 | 약전 | 적량 |

5.2.3. 저장방법

- 기밀용기, 냉장보관 (2~8℃)

**6. 임상시험 대상 환자군**

**6.1 대상 환자군**

- 고용량 항암화학요법을 예정하는 성인 악성혈액질환 환자 중 본 연구에 동의하는 자

**6.2 선정 기준**

1. 만 18세 이상의 성인
2. 악성혈액질환이 확진된 자
3. 고용량 항암화학요법을 예정하고 있는 입원 환자
4. ECOG performance status 0-2인 자 [별첨6]
5. 본 연구에 참여하고자 서면 동의한 자 [별첨1, 2]
   1. **제외 기준**
6. 본 약제 또는 이와 비슷한 계열의 약제에 과민성을 갖는 자
7. 항암화학요법 이전에 구강 내 궤양 또는 단순 포진이 있는 자. 즉, 중증도 평가도구- NCI CTCAE v3.0 기준 grade 0 이 아닌 경우
8. 중증의 치과 질환이 있는 자
9. 연구 참여 전 3주 이내에 항암화학용법 또는 방사선 치료를 받은 자
10. 연구 참여 전 3주 이내에 외과적 수술을 받은 자
11. 본 연구에 영향을 줄 수 있는 임상시험에 본 연구 참여 전 4주 이내에 참여하였거나 현재 참여 중인 자
12. 악성혈액질환 외에 연구에 영향을 미칠 만큼의 중증 질환은 동반하고 있는 자
13. 중증 정신질환자 또는 약물 및 알코올 중독 환자
14. 임신 중이거나 수유중인 여성
15. 기타, 연구자에 의해 본 연구에 수행이 곤란하다고 판단되는 자
16. 환자가 연구에 동의하지 않는 경우

**7. 연구기간**

- 2009.02.01 – 2010. 01.31

**8. 연구 방법**

- 1. **연구방법 개요**
- Double blind, Placebo-controlled, Randomized method, Single-center study

**8.2. 연구 진행**

8.2.1. 스크리닝기 (Screening period)

1. 항암화학요법 시행 전 7~1일
2. 연구 피험자 선정 및 평가
3. 기초 임상병리검사 시행
4. 환자 병력의 확인
5. 피험자 서면 동의서 획득

8.2.2. 투약기 (Administration period)

1. 항암화학요법 시작일로부터 골수기능 회복 시 (ANC가 1000이상으로 3일 이상 지속되는 경우)까지
2. 골수 기능 회복 후에도 구내염이 지속되는 경우 구내염 증상이 소실될 때까지 (WHO oral mucositis scoring system scale grade 0 기준)
3. 피험자 중도 탈락 또는 연구가 중지되는 경우 이전에 종료 가능


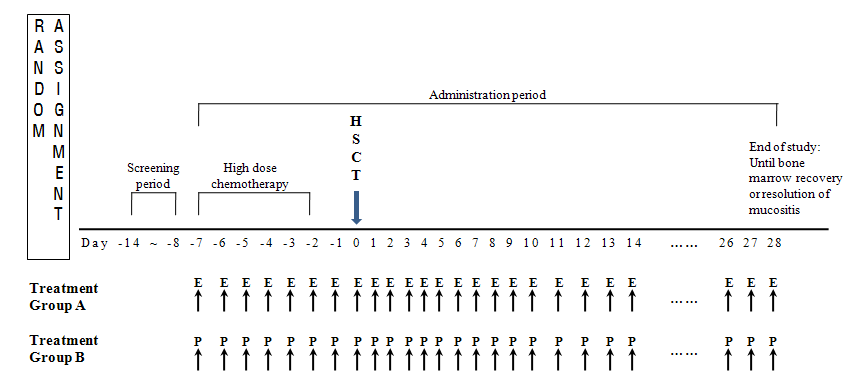


Fig 1. Study design and schedule. HSCT, Hematopoietic Stem Cell Transplantation; Treatment Group A, recombinant human epidermal growth factor (E); Treatment Group B, placebo (P).

**8.3. 목표 피험자의 수 및 산출 근거**

8.3.1. 목표 피험자 수

- 본 연구는 시험약군과 위약군을 1:1로 배정하고 탈락률을 10%로 고려하여 각 군 당 69명씩 총 138명을 목표 피험자수로 산정하였다.

8.3.2. 산출 근거

- 본 연구의 목적은 재조합 인간상피세포성장인자 외용액을 사용 한 경우와 위약을 사용한 경우를 비교하여 악성혈액질환 환자에서 고용량 항암화학요법에 의해 발생한 구내염에 재조합 인간상피세포성장인자 외용액이 효과가 있는지를 알아보는 것으로 다음을 가정하여 표본의 크기를 산출하였다. 연구 가설은 다음과 같다.
- 가설
- [귀무가설] 재조합 인간상피세포성장인자 외용액제 사용 유무에 따른 치료군과 대조군의 치료유효성은 차이가 없다.
- [대립가설] 재조합 인간상피세포성장인자 외용액제 사용 유무에 따른 치료군과 대조군의 치료유효성은 차이가 있다.
- 가정
- 최종 1종 오류(α, level of significance)=0.05
- 군 간 시험예수의 비율, 치료군: 대조군 = 1:1
- 검정력(Power of test)은 80%를 유지
- 방사선 치료로 인한 구내염에 대해 재조합 인간상피세포성장인자 외용액의 효과를 평가한 선행연구에서 “RTOG grade 2 이상(NCI CTCAE v3.0에서 grade 2 이상)의 구내염이 발생하지 않은 피험자 분율”에 대한 ITT 분석 결과 위약군의 분율은 37%, 재조합 인간상피세포성장인자 투여군은 64%였다. (투여약물의 용량 및 용법은 본 연구와 동일)
- 산출결과
- 피험자수는 군 당 62명이고, 탈락율 10%를 고려하면 전체 피험자 수는 군 당 69명 총 138명이 요구된다.

| **Numeric Results for Two-Sided Test of Proportions. Continuity Correction Applied.**  **Power N1 N2 Alpha Beta P1 P2**  0.806915 62 62 0.050000 0.193085 0.37 0.64 |
| --- |

**8.4. 비교군 설정**

- 본 연구는 여러 인자로부터 발생할 수 있는 변동성(variability)과 비뚤림(bias)을 예방하기 위해 위약을 투여하는 비교군을 설정하였다. 위약비교군을 설정함으로써 환자의 심리적 영향으로 인한 결과의 비뚤림을 배제할 수 있으며, 결과변수를 해석함에 있어 약물과의 인과성을 변별하는데 도움을 주어 분석결과의 신뢰성을 고취할 수 있다.
- 비교군에 위약을 투여하는 것에 대한 윤리적 문제는 현재 항암화학요법으로 인한 구내염에 인정되는 표준치료방법이 없으며 대증적 요법에 의존한다는 점과 본 연구에서 대증요법 중 하나인 클로르헥시딘, 베타딘, 니스타틴 가글을 사용한다는 점에서 허용 가능하다고 판단된다.

**8.5. 이중맹검법**

- 본 연구에서는 이중맹검을 유지하기 위해 시험약과 대조약 간의 용량, 색깔, 냄새, 맛, 포장 및 라벨링을 동일하게 하여 제공한다. 피험자는 무작위배정법에 따라 시험약군/대조약군으로 결정되며, 부작용 발생등의 경우를 제외하고는 연구 종료 시까지 어느 누구도 맹검을 해지할 수 없다.

**8.6. 무작위배정법**

- 본 연구에서는 군간 균형성을 위하여 시험약군과 위약군을 1:1의 비율로 무작위 배정한다. 무작위배정은 서울대학교병원 의학연구협력센터 홈페이지 (<http://mrcc.snu.ac.kr>)에 피험자 인적사항 및 선정기준을 입력하고 해당 프로그램에 따라 수행된다.

**8.7. 투여량, 투여방법, 투여기간**

8.7.1. 투여량

- 시험약 또는 위약을 1일 2회, 구강 점막 부위가 충분히 덮일 정도로 분사한다.

8.7.2. 투여방법

- 구강점막부위에 약물을 1회 투여 시, 입천장, 목젖, 양 뺨 안쪽, 혀, 입술 안쪽 각각의 부위에 1회씩 분사한다. 약물 투여 후 30분 동안은 구강섭취를 제한한다.

8.7.3. 투여기간

- 전체 투여기간은 항암화학요법 시작일로부터 골수기능 회복 시 (ANC가 1000 이상으로 3일 이상 지속되는 경우)까지 또는 골수 기능 회복 후에도 구내염이 지속되는 경우 구내염 증상이 소실될 때까지로 한다.

**8.8. 병용요법**

8.8.1. 허용 약물 및 처치

1. 구강 위생을 위해 클로르헥시딘(chlorhexidine), 베타딘(betadine) 및 니스타틴(nystatin) 가글을 사용 할 수 있다.
2. 조혈모세포이식의 부작용을 예방하기 위하여 ciprofloxacin, trimethoprim/sulfamethoxazole, fluconazole, heparin, intravenous immunoglobulin, parenteral granulocyte colony stimulating factor (G-CSF)와 같은 보조 약물을 투여할 수 있다.
3. 구내염으로 인한 통증에 대해서는 마약성/비마약성 진통제를 투여할 수 있다.

8.8.2. 금지 약물 및 처치

1. beta-carotene, silver nitrate 단일제, tocopherol 단일제, granulocyte colony stimulating factor (G-CSF) 또는 granulocyte macrophage colony stimulating factor (GM-CSF) gargling, 그 외 구내염 치료에 직접적으로 영향을 미칠 수 있다고 판단되는 약물.
2. 정해진 항암제 투여 이외의 다른 항암치료법

**8.9. 피험자 교육**

- 연구에 참여하는 모든 피험자는 종양전문간호사 및 연구약사로부터 항암화학요법 전에 약물사용, 예후, 이상반응 및 대처법, 일상생활 등에 대한 전반적 교육을 동일하게 받는다.
- 연구에 참여하는 모든 피험자는 연구담당자로부터 약품 보관과 투여방법 및 주의사항, 투약기록지 작성법 등에 대한 교육을 받는다.
- 기밀용기에서 냉장보관 (2~8℃)
- 투여 직전 거품이 나지 않을 정도로 가볍게 용기를 흔듦
- 투여 후 30분 동안 물이나 음식물을 섭취하지 않음
- 임의로 약물을 중단하지 않음

**8.10. 관찰 항목, 검사방법**

8.10.1. 중증도 평가

- 스크리닝기(1회)를 포함하여 항암화학요법이 시작되는 날부터 투약이 종료되는 때까지 매일 시행한다.
- 연구자가 피험자 구강 내 부위를 관찰하며, 두 가지 측정도구-NCI CTCAE v3.0, WHO oral mucositis scoring system scale-를 이용해 구내염의 중증도를 평가한다.

8.10.2. 구강과 목의 통증 및 연하 곤란에 대한 피험자 자가평가

- 피험자 자가평가는 항암화학요법이 시작되는 날부터 투약이 종료되는 날까지 매일 시행한다.
- 평가는 연구담당자가 통일된 방법으로 질문 시, 환자가 이에 응답하는 방법으로 시행하며, 측정도구로는 OMDQ (oral mucositis daily questionnaire)를 이용한다.

8.10.3. 의무 기록지

- 피험자의 마약성 진통제 사용 여부를 포함한 연구약물 외 모든 병용약물의 사용을 매일 검토하여 증례기록지에 기록한다.
- 피험자의 감염 발생 여부, 종류, 중증도, 지속기간, 치료약제 등에 대하여 증례기록지에 기록한다.
- 이식 후 발생하는 급성 이식편대숙주반응 (acute graft versus host disease, acute GvHD), 정맥폐쇄병 (veno-occlusive disease, VOD)의 발생여부, 중증도, 지속기간, 양상, 치료약제 등에 대하여 증례기록지에 기록한다.

8.10.4. 이상반응

- 시험약의 안전성 평가와 관련하여 매일 이상반응의 발생여부를 관찰한다.
- 이상반응이 발생한 경우에는 발생일, 종류, 중증도, 시험약과의 관련성, 시험약의 지속여부, 치료 등에 대하여 증례기록지에 기록하고 평가한다. [별첨9]
- 시험약과 관련하여 중대한 이상반응이 발생할 경우 보고서를 작성하여 임상시험책임자, 임상시험심사위원회 등에 보고한다. [별첨10]

8.10.5. 순응도(%) 평가 및 방법

- 순응도 평가는 항암화학요법이 시작되는 날부터 투약이 종료되는 날까지 매일 시행한다.
- 평가는 연구담당자가 통일된 방법으로 질문 시에 환자가 응답하는 방법으로 시행하며, 이 때 시험약 또는 위약의 남은 양을 비교하여 평가한다.
- 순응도(%) = 피험자의 실제 투여 회수

피험자가 정상적으로 투여해야 하는 회수

Table1. 연구수행 일정표

| 기간  연구내용 | 스크리닝기 | 투약기 | |
| --- | --- | --- | --- |
|  |  | 고용량 항암화학요법 시행기 | 이식 후 회복기 |
| 피험자 평가 및 선정 | O |  |  |
| 연구 목적 설명 | O |  |  |
| 피험자 서면 동의서 획득 | O |  |  |
| 병력 확인 | O |  |  |
| 신체검사 및 활력징후 | O |  |  |
| 임상병리검사 시행 | O |  |  |
| 무작위 배정 |  | O |  |
| 시험약/대조약 처방 |  | O |  |
| 피험자 교육 |  | O |  |
| 구내염 중증도 평가 | O | O | O |
| 통증에 대한 피험자평가 |  | O | O |
| 이상반응 모니터링 |  | O | O |
| 병용약물 모니터링 |  | O | O |
| 순응도 평가 |  | O | O |

**8.11. 안전성**

8.11.1. 이상반응과 실험실적 검사 이상치

1. 이상반응

- 이상반응(adverse event, AE)이란 환자나 시험약물을 복용한 임상시험의 피험자에서 나타난 부적절한 의학적 반응의 발생이며, 이는 치료와 반드시 인과관계를 가질 필요는 없다. 즉, 이상반응은 약물과의 관련여부에 상관없이 약물의 사용과 관련하여 일시적으로 나타나는 바람직하지 않고 의도하지 않은 징후(예, 비정상적 실험실 검사결과포함), 증상, 또는 질병을 말한다.
- 이상반응 발생 시에 중증도는 NCI-CTC, Version 3.0에 기초하여 등급을 정하며, 자세한 정보는 증례기록지에 기록한다. NCI-CTC grading 체계에 없는 이상반응의 경우, 경증, 중등증, 중증, 생명의 위협, 사망의 5개 등급으로 분류한다. 이러한 이상반응과 치료간의 인과관계 평가가 필요하다 [별첨7].
- 1도 (경증 이상반응)
- 2도 (중등증 이상반응)
- 3도 (중증 이상반응)
- 4도 (생명을 위협하는 또는 불구를 가져오는 이상반응)
- 5도 (이상반응과 관련된 사망)

1. 중대한 이상반응 정의

- 중대한 이상반응은 임의의 용량에서 다음과 같은 결과를 초래하는 바람직하지 않은 의학적 사건이다.
- 사망의 초래
- 즉각적인 생명의 위협 (주. ‘생명의 위협(life-threatening)’이라는 용어는 사건이 발생한 시점에 환자가 사망의 위험에 처해있는 경우를 가리키며, 보다 중증으로 나타났더라면 사망을 초래했을지도 모른다는 가정적 사건을 의미하지는 않는다.)
- 입원 혹은 입원기간의 연장이 필요
- 영구적인 혹은 유의한 불구/기능저하의 초래 (주. ‘불구’는 일상생활기능을 수행하는 환자의 능력에 유의한 저하를 의미한다.)
- 선천적 기형/출생 시 결함
- 의학적 판단에 따른 중요한 의학적 사건 (환자를 위태롭게 하거나 위에 열거된 다른 결과를 예방하기 위하여 내과적 또는 외과적 중재를 필요로 하는 상황 (예, 입원을 초래하지 않은 알러지성 기관지 연축, 경련, 무증상의 ALT 증가>10xULN, 약물의존성, 약물남용))

1. 실험실검사결과 비정상

- 실험실검사결과 비정상은 그 자체로는 증례기록지의 이상반응 페이지에 기록하기 위한 이상반응으로 보고되지 않는다. 그러나, 아래 경우 중 하나에 해당되면 이상반응으로 보고한다.
- 환자가 비정상 결과로 치료를 받거나 결과로 인한 동반치료의 변화와 관련된 임상상태를 보이는 경우
- 시험약 투여가 중지/지연됨에도 불구하고 중대한 이상반응으로 간주되는 경우; 시험약 용량이 변화된 경우
- 시험약 투여가 영구 중단된 경우.

8.11.2. 이상반응 보고절차

1. 이상반응 공지 및 기록

- 모든 이상반응은 환자 차트 및 증례기록지에 기록하고 보고해야 한다.

1. 중대한 이상반응 공지 및 기록

- 임상시험 중 중대한 이상반응 발생시 각 담당자의 의무는 다음과 같다.
- 임상시험 책임자의 의무
- 임상시험 기간 동안 또는 치료 후 기간 동안 중증 또는 의학적으로 중요한 임상적 이상반응이나 실험실검사수치가 발생하면, 피험자가 받은 치료에 관계없이 시험자는 책임연구자와 임상시험심사위원회(IRB)에 즉시 보고한 후 시험자가 알게 된 날로부터 1주일 이내에 자세한 서면보고를 해야 한다. 단, 사망을 초래하거나 생명을 위협하는 경우에는 3일 이내에 추가보고를 하여야 한다. 이 경우 즉시 및 상세 보고에는 피험자의 신원을 보호하기 위하여 피험자의 성명, 주민등록번호 및 주소를 기재하는 대신 피험자식별코드를 사용하여야 한다. 사망 예를 보고한 경우 책임연구자는 심사위원회에게 부검보고서(부검을 실시한 경우에 한함)와 사망진단서 등의 추가적인 정보를 제공하여야 한다.
- 시험자는 관련법률에 따라 모든 보고요건을 따르는지 확인해야 한다.
- 시험자는 피험자의 사망을 보고하는 경우, 책임연구자, IRB에 요청 받은 부가정보를 제공해야 한다.
- 임상시험심사위원회(IRB)의 의무
- 임상시험심사위원회는 예상하지 못한 중대한 이상약물반응이나 피험자의 안전성이나 임상시험의 실시에 부정적인 영향을 미칠 수 있는 새로운 정보에 관한 사항이 발생한 경우 필요한 조치를 임상시험 책임자에게 하여야 한다.

1. 이상반응을 가진 피험자 모니터링

- 모든 이상반응(이의 치료들)은 시험기간 동안, 마지막 용량 투여 후 28일 동안 기록되어야 한다. 환자는 이상반응이 해결되거나 피험자가 시험약 투여를 완료한 후에도 타당한 설명이 가능할 때까지 추적관찰 되어야 한다. 또한 SAE는 SAE 문서로 보고하여야 한다. 모든 필요한 추가 치료 조치와 추적 절차를 시행하는 것은 시험자의 책임이다.

1. 과용 및 중독

- 현재 시험 중 약물과용이 발생하는 경우, 이는 SAE로 보고해야 한다.

1. 임신

- 환자가 시험기간 동안 임신하게 되면, 환자는 시험약 투여를 중단하고 즉시 시험자에게 이를 알리도록 교육받아야 한다. 또한 시험약 투여완료 후 90일 이내 발생하는 모든 임신은 시험자에게 보고되어야 한다. 시험자는 환자의 결과를 포함하는 추적 보고와 함께 24시간 이내 IRB에 모든 임신을 보고하도록 한다. 시험자는 임신기간 동안 태아에 미치는 위험 및 가능한 영향에 대해환자와 상의해야 하며, 임신결과를 알 때까지 환자를 모니터 해야 한다. 환자는 시험약 투여를 중지하고 추적기를 갖게 된다.
- 시험에 참여하는 환자의 배우자가 임신하는 경우, 이는 시험자에게 보고되어야 한다. 시험자는 배우자와 상담하며, 위의 설명한 지침을 따라야 한다.

**8.12. 예측 부작용 및 사용상 주의사항**

8.12.1. 예측 부작용

- 당뇨병성 족부궤양 환자를 대상으로 한 이지에프외용액 0.005%의 제2상 임상시험의 결과에서 시험약과의 관련성이 “possible”로 판정된 이상반응으로는 분무부위의 통증이 있었고, “unknown”으로 판정된 이상반응으로는 궤양부위의 overgrowth, GOT/GPT 상승, 설사가 있었다. 또한 창상 환자를 대상으로 한 제 2상 임상시험 결과에서는 시험약 및 대조약과 관련성이 있는 이상약물반응은 한 건도 발생하지 않았다. 사용상의 주의사항 및 예측되는 이상반응에 대해서는 매 평가 시에 주의해서 평가하도록 한다.

8.12.2. 사용상 주의사항

- 다음 환자에는 투여하지 말 것
- 동 성분에 과민증이 있는 환자
- 일반적 주의
- 정해진 용법·용량을 지켜야 한다.
- 어린이에 투여할 경우에는 보호자의 지도·감독 하에 투여한다.
- 사용 후 과증식(overgrowth) 현상이 나타날 수 있다.
- 임부·수유부에 대한 투여
- 임신 중 투여에 대한 안전성이 확립되어 있지 않다.
- 보관 및 취급상의 주의
- 사용 후 반드시 2∼8 ℃의 냉장실에 보관할 것
- 어린이의 손이 닿지 않는 곳에 보관할 것
- 기타
- 시험관내 시험에서 면역기능 증가를 나타내는 것으로 보고되었다.

**9. 평가 변수 및 통계분석**

**9.1. 평가변수**

9.1.1. 주 평가변수

- 투약기간 동안 NCI CTCAE v3.0 기준 grade 2 이상의 구내염이 발생하지 않은 피험자 분률

9.1.2. 부 평가변수

- 투약기간 동안 NCI CTCAE v3.0 기준 grade 2 이상의 구내염 발생까지의 기간 및 지속기간
- 투약기간 동안 NCI CTCAE v3.0 기준 grade 3 이상의 구내염 발생률, 발생까지의 기간 및 지속기간
- 투약기간 동안 WHO oral mucositis scoring system scale 기준 grade 2 이상의 구내염 발생률, 발생까지의 기간 및 구내염 지속기간
- 투약기간 동안 WHO oral mucositis scoring system scale 기준 grade 3 이상의 구내염 발생률, 발생까지의 기간 및 구내염 지속기간
- 투약기간 동안 WHO oral mucositis scoring system scale 기준 grade 4 이상의 구내염의 발생률, 발생까지의 기간 및 지속기간
- 투약기간 동안 OMDQ (oral mucositis daily questionnaire) score
- 구내염으로 인한 마약성 진통제 총 투여 일수
- 재조합 인간상피세포 성장인자의 부작용 및 안전성 평가
- 이상반응

**9.2. 통계분석**

9.2.1. 환자 기초 자료 분석방법

- 시험약군과 비교군 피험자의 기초 자료에 있어 동질성을 검정한다.
- 일변량 분석으로 연속형 자료의 경우 t-test 또는 Mann Whitney U test를 시행하고, 이분형 자료의 경우 Chi-square 또는 Fisher’s exact test 등을 시행한다.

9.2.2. 결과 분석 원칙

- 주 평가변수와 부 평가변수에 대한 결과 분석은 시험에 참여한 초기 피험자 모두를 대상으로 하는 intention to treat (ITT) 분석을 기본으로 하며, ITT 분석군 중에서 임상시험을 완료하였으며 순응도 75% 이상인 피험자에 대해 per protocol (PP) 분석을 병행한다.

9.2.3. 유효성 분석

1. 주 평가변수

- 본 연구는 항암화학요법에 의해 발생한 구내염에 재조합 인간상피세포성장인자가 효과가 있는지를 기존의 치료법에 대해 비교하고자 하는 것으로 주 평가변수는 투약기간 동안 NCI CACAE v3.0 기준 grade 2 이상 구내염이 발생하지 않은 피험자의 분율이다.
- 따라서 다음의 가설검정을 시행할 수 있다.

n_1_: 비교군 피험자 수 n_2_: 시험약군 피험자 수

x_1_: 비교군에서 grade 2 이상 구내염이 발생하지 않은 피험자 수

x_2_: 시험약군에서 grade 2 이상 구내염이 발생하지 않은 피험자 수

연구 가설 H_o_: P_2_ – P_1_ = 0

H_A_: P_2_ – P_1_ ≠ 0

검정통계량

1. 부 평가변수

- 부 평가변수의 특성에 따라 Chi-square test 또는 Fisher’s exact test, T-test 또는 Mann Whitney U test 등 적절한 검정방법을 이용하여 시험약군과 비교군을 평가한다.

9.2.4. 안전성 분석

- 이상반응 발생자 수, 발생 건수를 산출하며 각 신체기관 별로 시험약과의 관련성, 중증도에 따라 분류하여 평가한다.

9.2.5. 중간 분석 (Interim Analysis)

- 중간분석의 일반적 원칙
- 본 임상시험에서는 총 2회의 통계분석을 실시한다. 중간분석은 목표한 피험자 수 138명 중 50%(69 명)에서 1차 평가가 완료되었을 때 실시하며 최종분석은 138명에 대해 평가가 완료된 후에 실시한다. 중간분석은 group sequential method 중 Lan and DeMets(1983)가 제안한 O’Brien-Fleming 타입의 alpha spending function 방법을 이용한다.

| **Details when Spending = O'Brien-Fleming, N1 = 62, N2 =62, P1 = 0.37, P2 = 0.64,**  **Continuity Correction.**  **Lower Upper Nominal Inc Total Inc Total**  **Look Time Bndry Bndry Alpha Alpha Alpha Power Power**  1 0.50 -2.96259 2.96259 0.003051 0.003051 0.003051 0.168499 0.168499  2 1.00 -1.96857 1.96857 0.049002 0.046949 0.050000 0.638415 0.806915 |
| --- |

- 중간분석의 일반적 원칙
- 중간분석에 사용될 유효성 평가변수는 1차 유효성 평가변수인 투약기간 동안 NCI CTCAE v3.0 기준 grade 2 이상의 구내염이 발생하지 않은 피험자 분율이다.
- 중간분석의 안전성 평가
- 안전성 분석은 재조합 인간상피세포성장인자를 1회 라도 투여 받은 모든 환자에 대해 이상반응 발생율을 산출한다.
- 중간분석 실시기준
- 1차 유효성 평가인 위약 군과 EGF 외용액제 군의 투약기간 동안 NCI CTCAE v3.0 기준 grade 2 이상의 구내염이 발생하지 않은 피험자 분율을 비교하기 위하여 카이제곱검정을 사용한다. 본 임상시험에서는 최종분석을 포함하여 두 번의 통계분석이 실시된다. 위에 언급된 중간분석 방법에 근거하여 두 번의 통계 검정에 대한 유의수준이 5%로 유지되도록 하기 위하여 중간분석에 사용될 유의수준은 0.003이고 최종분석에 사용될 유의수준은 0.047 이다.

**10. 피험자 보상에 관한 규약**

- 본 임상시험에 참여하는 피험자는 입원기간 동안 임상시험용 의약품을 무료로 제공받는다.
- 환자가 별도로 본 연구를 위해 병원을 방문해야 하는 것이 아니라 입원기간 동안 연구가 진행되므로 임상시험용 의약품 제공 외에 별도의 보상비용이 필요치 않을 것으로 사료된다.
- 본 임상연구에 참여하는 피험자는 본 임상연구와 관련 된 손상이 발생하였을 경우, “피험자보상에 대한 규약”의 내용에 근거하여 적절한 보상을 받는다. [별첨10]

**11. 피험자의 인권 및 윤리 문제**

**11.1. 윤리원칙**

본 임상시험은 서울대학교병원에서 시행하는 임상연구로 모든 참여 시험자는 임상연구가 프로토콜과 헬싱키 선언 (World Medical Association Declaration of Helsinki, current revision) 및 1996년 7월 17일에 승인된 International Conference on Harmonization of Technical Requirements of Pharmaceuticals for Humans Use (ICH) Note for Guidance on Good Clinical Practice (ICH, Topic E6, 1995)에 기초하는 윤리적 원칙과 해당되는 규제 요구사항에 따라서 시행됨을 보증할 의무가 있다. 이러한 문서들은 피험자의 서면 동의서가 임상 시험 참여의 핵심적인 선행조건임을 명시하고 있다. 연구의 시작에 앞서 연구 프로토콜은 관련된 문서들(환자 설명문, 동의서 양식, 시험자용 책자)과 함께 해당되는 IRB/EC에 승인을 위하여 제출된다. IRB/EC의 승인은 연구파일에 보관될 것이다. 연구는 서면 승인서를 획득한 후에만 진행될 것이다. 프로토콜의 모든 수정사항은 IRB/EC에 제출될 것이며, IRB는 국내 및 지방 규정에 따라서 SAE에 대하여 통보될 것이다.

**11.****2. 피험자 설명**

피험자의 연구참여에 있어서 무조건적인 선행 조건은 환자의 서면 동의이다. 따라서, 서면 동의를 받기 전에 시험자는 피험자에게 적절한 설명을 제공하여야 한다. 서면 동의의 획득을 위하여 지방 언어로 기술된 ICH Note for Guidance on Good Clinical Practice (ICH, Topic E6, 1995)에 부합하는 환자 설명문을 제공한다. 서면 설명문과 함께 시험자 혹은 위임 받은 자는 피험자에게 구두로 설명을 제공한다. 설명함에 있어서 일반인도 충분히 바로 이해할 수 있는 단어를 선택하도록 한다. 환자 설명문은 환자 동의에 있어서 중요한 새로운 정보가 입수되는 경우 언제든지 수정되어야 한다.

**11.****3. 피험자 동의**

피험자의 연구 참여에 관한 서면 동의가 있은 후에만 연구와 관련된 어떠한 행위도 시행될 수 있다. 동의서는 피험자가 직접 서명하고 날짜를 기입하여야 하며 시험자 혹은 위임 받은 자가 동의에 관하여 토의하여야 한다.

동의서의 획득은 시험자에 의하여 증례기록지에 확인되어야 한다. 날짜가 기입된 서명 동의서는 시험자가 안전하게 보관하여서 감시, 감사, 시찰의 목적으로 언제든지 검색할 수 있어야 한다. 날짜가 기입된 서명 동의서의 사본은 연구 참여 전에 피험자에게 교부되어야 한다.

만약 피험자 혹은 법적으로 수용 가능한 대리인이 읽을 수 없는 경우에는 신뢰할 수 있는 독립적인 입회자가 동의에 관한 토의과정 전반에 참석하여야 한다. 입회자의 선택은 피험자의 비밀의 권리를 침범하지 않게 선택되어야 한다. 신뢰할 수 있는 독립적인 입회자는 해당 기관에 소속되지 않으며 연구에 관여하지 않는 사람으로 정의된다. 가족 구성원이나 지인이 적적한 독립적인 입회인이다. 피험자 혹은 법적으로 수용 가능한 대리인이 구두로 동의하고 가능하면 서명한 후에, 입회인은 정보가 정확하고 피험자 혹은 법적으로 수용 가능한 대리인이 동의서의 동의 조항을 완벽히 이해하였고 진정한 동의를 시행하였음을 증명하는 동의서에 직접 날짜를 기입하고 서명하여야 한다.

**11.4. 피험자에 대한 안전성의 배려**

임상시험 연구자는 본 임상시험계획서에 명시된 이상반응 및 주의사항 등에 대하여 사전에 충분히 숙지하고 임상시험 진행도중 면밀히 관찰한다. 시험 도중 중대한 이상반응 등이 발생한 경우에는 즉시 보고하며, 임상시험의 계속 또는 중지여부는 임상시험심사의원회의 결정에 따른다. 또한 임상시험 실시기관은 본 임상시험계획서에 규정된 대로 임상시험이 적절히 진행될 수 있도록 임상시험에 필요한 설비와 전문인력을 갖추고, 해당 임상시험을 적절하게 실시할 수 있도록 준비에 완벽을 기하도록 한다.

**11.5. 피험자의 정보 보호**

본 연구와 관련된 정보는 순수 연구 목적으로만 사용 될 것이며 모든 자료는 엄격하게 환자의 비밀이 유지되어 보호될 것이다. 데이터 파일은 서울대학교병원 암센터 자료 분석실의 데이터 보관 장소에 보관될 것이며 자료 분석실은 연구자 이외의 접근이 허용되지 않는 잠금장치가 있다. 환자의 개인 정보 보호를 위하여 환자의 신상을 알 수 있는 정보와 환자에 관한 정보는 독립적인 데이터로 관리 될 것이다.

**12. 연구의 기대 성과**

구강점막의 상피세포를 포함한 다양한 조직세포의 분화 및 성장을 촉진시킨다고 알려져 있는 재조합 인간상피세포성장인자는 지금까지의 선행연구 결과들로부터 구내염에 대한 치유효과 및 안전성을 나타낼 수 있을 것으로 기대된다. 따라서 고용량 항암화학요법을 시행하는 악성혈액질환 환자에서 재조합 인간상피세포성장인자의 유효성 및 안전성에 대하여 연구함으로써, 이들 환자의 구내염의 효과적인 예방 및 치료법으로서 재조합 인간상피세포성장인자의 역할을 제시하고자 한다.

**13. 참고문헌**

1. NCI. Oral Complications of Chemotherapy and Head/Neck Radiation (PDQ): Health Professional Version. 2005
2. [Trotti A](http://www.ncbi.nlm.nih.gov/sites/entrez?Db=pubmed&Cmd=Search&Term=%22Trotti%20A%22%5BAuthor%5D&itool=EntrezSystem2.PEntrez.Pubmed.Pubmed_ResultsPanel.Pubmed_DiscoveryPanel.Pubmed_RVAbstractPlus), [Bellm LA](http://www.ncbi.nlm.nih.gov/sites/entrez?Db=pubmed&Cmd=Search&Term=%22Bellm%20LA%22%5BAuthor%5D&itool=EntrezSystem2.PEntrez.Pubmed.Pubmed_ResultsPanel.Pubmed_DiscoveryPanel.Pubmed_RVAbstractPlus), [Epstein JB](http://www.ncbi.nlm.nih.gov/sites/entrez?Db=pubmed&Cmd=Search&Term=%22Epstein%20JB%22%5BAuthor%5D&itool=EntrezSystem2.PEntrez.Pubmed.Pubmed_ResultsPanel.Pubmed_DiscoveryPanel.Pubmed_RVAbstractPlus), et al. Mucositis incidence, severity and associated outcomes in patients with head and neck cancer receiving radiotherapy with or without chemotherapy: a systematic literature review. [Radiother Oncol.](javascript:AL_get(this,%20'jour',%20'Radiother%20Oncol.');) 2003 Mar;66(3):253-62
3. Edward BR, Douglas EP, Mark S, et al. Clinical Practice Guidelines for the Prevention and Treatment of Cancer Therapy–Induced Oral and Gastrointestinal Mucositis. Cancer 2004;100(9 Suppl):2026–46.
4. Paris F, Fuks Z, Kang A, et al. Endothelial apoptosis as the primary lesion initiating intestinal radiation damage in mice. Science Jul 2001;293(5528):293-97
5. Sonis ST, Elting LS, keefe D, et al. Perspectives on cancer therapy-induced mucosal injury. Cancer 2004;100(9):1995-2025
6. Sonis ST. Pathobiology of oral mucositis: novel insights and opportunities. [J Support Oncol.](javascript:AL_get(this,%20'jour',%20'J%20Support%20Oncol.');) 2007 Oct;5(9 Suppl 4):3-11.
7. Sonis ST, Oster G, Fuchs H, et al. Oral mucositis and the clinical and economic outcomes of hematopoietic stem-cell transplantation. J Clin Oncol. 2001;19:2201–05.
8. Dorothy MK, Mark MS, Linda SE, et al. Updated Clinical Practice Guidelines for the Prevention and Treatment of Mucositis. Cancer 2007;109:820–31.
9. Inger von B, Michael TB, Fred KLS, et al. Growth factors and cytokines in the prevention and treatment of oral and gastrointestinal mucositis. Support Care Cancer 2006;14: 519–27
10. Cohen S. Isolation of a mouse submaxillary gland protein accelerating incisor eruption and eyelid opening in the newborn animal. J Biol Chem 1962;237:1555–1562.
11. Nanney LB, King LE Jr., Dale BA. Epidermal growth factor receptors in genetically induced hyperproliferative skin disorders. Pediatr Dermatol 1990;7:256 –265.
12. Servold SA. Growth factor impact on wound healing. Clin Podiatr Med Surg 1991;8:937–953.
13. Ino M, Ushiro K, Ino C, et al. Kinetics of epidermal growth factor in saliva. Acta Otolaryngol 1993;500:126–130
14. Brown GL, Curtsinger L 3rd, Brightwell JR, et al. Enhancement of epidermal regeneration by biosynthetic epidermal growth factor. J Exp Med 1986;163:1319 –1324.
15. Brown GL, Curtsinger LJ, White M, et al. Acceleration of tensile strength of incisions treated with EGF and TGF-beta. Ann Surg 1988;208:788 –794.
16. Sonis ST, Costa JW, Jr., Evitts SM, et al. Effect of epidermal growth factor on ulcerative mucositis in hamsters that receive cancer chemotherapy. Oral Surg Oral Med Oral Pathol 1992;74:749-55.
17. Wu-Wang CY, Patel M, Feng J, et al. Decreased levels of salivary prostaglandin E2 and epidermal growth factor in recurrent aphthous stomatitis. Arch Oral Biol 1995;40:1093-8.
18. Dumbrigue HB, Sandow P, Nguyen KH, et al. Salivary epidermal growth factor levels decrease in patients receiving radiation therapy to the head and neck. Oral Surg Oral Med Oral Pathol 2000;89:710–16
19. Epstein JB, Emerton S, Guglietta A, et al. Assessment of epidermal growth factor in oral secretions of patients receiving radiation therapy for cancer. Oral Oncol 1997;33:359–63
20. Epstein JB, Gorsky M, Guglietta A, et al. The correlation between epidermal growth factor levels in saliva and the severity of oral mucositis during oropharyngeal radiation therapy. Cancer 2000;89:2258-65.
21. 우홍균 외. 방사선 구강점막염에 대한 상피세포성장인자의 효과를 보기 위한 파일럿임상시험 결과. 대한방사선종양학회지 2006.
22. JP HONG, SW LEE, SY SONG, et al. Recombinant human epidermal growth factor treatment of radiation- induced severe oral mucositis in patients with head and neck malignancies. European Journal of Cancer Care 2008.
23. Y. Ahn, H. Wu, S. Song, et al. The therapeutic effect of recombinant human epidermal growth factor (rhEGF) on mucositis in patients with head and neck cancer undergoing radiotherapy with or without chemotherapy: a double-blind placebo-controlled prospective phase II multi-institutional study. J Clin Oncol 26: 2008 (May 20 suppl; abstr 6021)

별첨1. 환자와 보호자를 위한 설명서

| **악성혈액종양질환 환자에서 고용량 항암화학요법에 의해 발생하는 구내염에 대한 재조합 인간상피세포성장인자의**  **무작위 2상 임상 연구** |
| --- |

안녕하십니까?

귀하께서는 신약을 이용하는 임상 연구에 참여하도록 요청을 받으셨습니다. 이 연구에는 참여를 원하시는 환자분만 참여하시게 됩니다. 귀하께서는 이 연구에 참여하시기 전에, 연구의 절차에 대한 다음의 설명을 읽고 이해하시는 것이 중요합니다.

**연구의 배경 및 목적:**

구내염은 고용량 항암제 전처치를 받는 조혈모세포 이식 환자의 약 75~85%에서 동반된다고 알려져 있습니다. 주로 항암치료 시작 후 4~5일부터 시작되며, 백혈구 감소증이 동반되지 않은 경우 보통 2~3주 사이에 치유되나 조혈모세포 이식 환자에서는 그 정도가 심하고 기간이 더 길다고 알려져 있습니다. 구내염의 주요 증상으로는 궤양, 발적, 부종, 통증, 미각상실, 구강건조증 등이 있습니다. 이러한 구내염은 암환자가 항암치료 중 가장 고통스럽게 경험하는 부작용 중 하나로 심한 통증 자체도 큰 문제가 되지만, 통증으로 인해 식사를 하기 어렵게 되어 삶의 질을 떨어뜨리고, 입원기간이 장기화되는 문제를 야기할 수 있습니다.

그 동안 구내염의 치료제에 대한 많은 연구가 이루어졌지만, 조혈모세포 이식 환자를 대상으로 효과가 입증된 치료제는 아직 ‘팔리페르민(palifermin)’이라는 약제 1가지뿐인데, 매우 고가(1주기당 1300만원, 70kg 성인 기준, 환율 1300원 기준)이며 현재 우리나라에 공식적으로 수입되지 않는다는 문제가 있습니다. 상기약제를 제외하고는 항암치료 과정 중 발생하는 구내염에는 아직까지 효과적으로 증명된 예방 및 치료법이 거의 없어서, 환자들은 주로 통증감소를 위한 국소마취제 및 마약/비마약성 진통제, 구강세척요법 등의 대증적 요법에 의존하고 있습니다.

최근 우리나라 6개 대학병원에서 방사선요법을 시행 받은 113명의 두경부암 환자를 대상으로 재조합인간상피세포 성장인자를 국소도포하고 효과와 이상반응을 보는 연구가 이루어졌는데, 구내염의 예방에 있어 유의한 효과를 나타내었고 중대한 이상반응은 발견되지 않았습니다. 이에 이번에 그 후속으로 조혈모세포 이식 환자에서 발생하는 구내염에 대하여 재조합 인간상피세포성장인자를 국소도포하여 그 예방효과, 치료효과 및 이상반응에 대한 연구를 시행하고자 합니다.

**연구용 약물:**

재조합 인간상피세포 성장인자는 각종 상피세포의 재생 및 치유 작용을 하는 것으로 체내에 정상적으로 존재하는 인자입니다.

재조합 인간상피세포 성장인자(상품명: 이지에프외용액 0.005%)는 당뇨병성 족부 궤양의 치료제로 이미 한국 식품의약품 안전청(KFDA, 2005년)의 허가를 받고 시판중인 약물입니다.

**연구용 약물 기대효과:**

재조합 인간상피세포 성장인자(이지에프외용액 0.005%)를 이용하여 두경부암 환자에서 방사선 치료로 인해 발생하는 구내염에 대한 유효성 및 안전성을 평가하는 선행연구가 있었는데, 유의한 예방 효과가 나타났으며 중대한 이상약물반응은 발생하지 않았습니다.

**부작용:**

현재까지 진행된 임상연구에서 중대한 이상약물반응은 발견되지 않았습니다. 그러나 현재까지 알려지지 않은 이상약물반응이 존재할 수 있습니다. 의료진과 연구진은 본 연구 대상 환자에서 이상약물반응의 발견 및 치료에 최선을 다할 것입니다.

**연구 방법:**

본 연구는 고용량 항암화학요법을 예정하고 있는 성인 입원 환자를 대상으로 연구의 조건에 적합하며 연구 참여에 자발적으로 동의한 환자를 대상으로 합니다. 연구 참여자는 무작위로 연구약군 또는 위약군에 배정받게 되며 각 군에 배정될 확률은 동일합니다. 연구약 또는 위약은 항암화학요법이 시작되는 날로부터 골수기능이 회복되는 날까지 (절대호중구수가 1000 이상으로 3일 이상 지속되는 경우) 또는 골수기능이 회복한 후에도 구내염이 있다면 구내염의 증상이 소실될 때까지 매일 1일 2회 투여합니다. 투여 시는 구강점막 부위가 충분히 덮일 정도의 양을 사용해야 하며, 약물 투여 후 약 30분 동안은 물이나 음식물의 섭취를 피해야 합니다.

연구기간 동안 환자 분은 매일 의료진으로부터 구강 상태를 확인 받으며 구내염 발생 시에는 구내염의 중증도와 통증 등을 평가 받습니다. 또한 환자분은 구강과 목의 통증 및 연하곤란에 대해 항암화학요법이 시작되는 날부터 투약이 종료되는 날까지 매일 의료진의 질문에 답하는 방식으로 자가평가를 하게 됩니다.

본 연구의 예상 참여인원은 총 138명(연구약군 69명, 위약군 69명)이며, 각 참여자의 예상 참여기간은 1개월 내외입니다.

**임상 연구 참여에 따른 혜택:**

본 임상연구에 참여하는 피험자는 입원기간 동안 임상시험용 의약품을 무료로 제공받습니다.

**피험자보상에 대한 규약:**

본 임상연구에 참여하는 피험자는 본 임상연구와 관련 된 손상이 발생하였을 경우, “피험자보상에 대한 규약”의 내용에 근거하여 적절한 보상을 받을 수 있습니다.

**자발적 참여:**

이 연구에의 참여 여부는 환자분의 자발적 의사에 따라 결정되며, 연구에 참여하지 않으시더라도 환자분은 어떠한 불이익도 받지 않습니다. 귀하는 언제든지 연구에 참여하지 않기로 결정할 수 있고 연구 참여 도중에도 중단할 수 있습니다. 이러한 귀하의 결정은 향후 진료에 영향을 미치지 않습니다.

**임상연구의 중도탈락:**

임상연구 참여와 지속여부는 전적으로 환자분의 결정에 따릅니다. 그러나 환자분이 참여 및 지속을 원하더라도 다음의 경우는 참여가 제한됩니다.

1. 선정 기준에 적합하지 않은 경우
2. 환자가 동의를 철회한 경우
3. 연구 기간 동안 연구 결과에 영향을 미칠 수 있는 약물의 처치 및 치료 등을 받은 경우
4. 연구 약물의 투약 순응도가 낮은 경우 (연속 2일 이상 투약 중단 시)
5. 구내염 중증도 평가가 누락된 경우 (연속 3일 이상 누락 시)
6. 심각한 이상반응으로 연구를 지속하기 어려운 경우

**비밀보장:**

연구에 참여하는 환자는 개인기록, 의무기록, 검사결과, 건강정보 등을 연구용 목적으로 제공하게 됩니다. 이러한 정보는 연구 진행 및 결과 도출을 위한 목적으로만 사용되며 연구 책임의사, 연구진행 담당자 및 병원 내 임상시험위원회에서 볼 수 있습니다.

단, 환자를 구분할 수 있는 자료는 기재되지 않고 비밀로 보장됩니다. 차후 학술적 논문 발표 시에도 모든 자료는 가공, 분석된 후 통합된 형태로 발표되므로 개인 신상 정보는 비밀로 유지됩니다.

**이상과 같은 사실을 숙지하시고, 고려하여 본 연구에 참여하실 것인지 아닌지를 결정해 주시기 바랍니다. 본 연구 또는 시험 참여자의 권익에 관해 추가적인 정보를 얻고자 하거나 연구와 관련한 문제가 발생한 경우 언제라도 연락을 주시기 바랍니다.**

서울대학교병원 혈액종양내과

책임연구자 윤성수

별첨2. 연구 참여 동의서

**연구 참여 동의서**

| **악성혈액질환 환자에서 고용량 항암화학요법에 의해 발생하는**  **구내염에 대한 재조합 인간상피세포성장인자의**  **무작위 2상 임상 연구** |
| --- |

본인은 고용량 항암화학요법에 의해 발생하는 구내염에 대해 재조합 인간상피세포 성장인자를 투여하는 임상연구에 있어 예상되는 효과 및 부작용 등에 대해 충분한 설명을 듣고 이해하였습니다.

이에 자발적인 의사로 본 임상연구의 참여에 동의합니다.

**200 년 월 일**

| **환자 또는 법적 대리인** | 성명  서명  환자와의 관계  날짜 | :  :  :  : |
| --- | --- | --- |
|  |  |  |
| **임상연구 담당자** | 성명  서명  날짜 | :  :  : |
|  |  |  |

별첨3. NCI Common Terminology Criteria for Adverse Events v3.0 (CTCAE)

| Grade 0 | Grade 1 | Grade 2 | Grade 3 | Grade 4 |
| --- | --- | --- | --- | --- |
| Normal | Erythema of the mucosa | Patchy ulcerations or  pseudomembranes | Confluent ulcerations or  pseudomembranes;  bleeding with minor  trauma | Tissue necrosis;  significant spontaneous  bleeding; life-threatening  consequences |

Reference) Cancer Therapy Evaluation Program, Common Terminology Criteria for Adverse Events, Version 3.0, DCTD, NCI, NIH, DHHS

별첨4. WHO Toxicity Criteria - Oral mucositis scoring system scale

| Grade 0 | Grade 1 | Grade 2 | Grade 3 | Grade 4 |
| --- | --- | --- | --- | --- |
| Normal | Erythema and soreness | Ulceration, but can eat solid foods | Ulceration, diet limited to liquids | Ulceration of such severity that patient requires parenteral feeding |

Reference) WHO handbook for reporting results for cancer treatment.

별첨5. OMDQ (oral mucositis daily questionnaire)

| Q1. 지난 하루 동안 환자분의 전체적인 건강 상태를 점수로 표현한다면 다음 중 몇 점에 해당합니까?  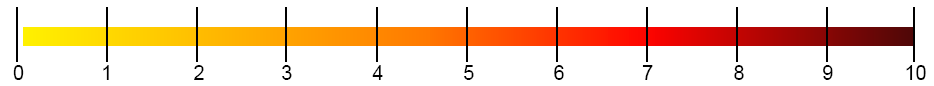  가장  좋음  가장  나쁨  Q2. 지난 하루 동안, 구강과 목의 통증은 다음 중 어느 정도입니까?   \| 통증 없음 \| ------------------------------------------------------ \| 0 \| \| --- \| --- \| --- \| \| 약간의 통증 \| ------------------------------------------------------ \| 1 \| \| 중간 정도의 통증 \| ------------------------------------------------------- \| 2 \| \| 상당히 심한 통증 \| ------------------------------------------------------ \| 3 \| \| 극도의 통증 \| ------------------------------------------------------ \| 4 \|   Q3. 지난 하루 동안, 구강과 목의 통증으로 다음의 활동이 어느 정도 제한을 받았습니까?   \|  \| 제한 없음 \| 약간의 제한이 있음 \| 다소 제한이 있음 \| 심한 제한이 있음 \| 통증으로 할 수 없음 \| \| --- \| --- \| --- \| --- \| --- \| --- \| \| 1. 침 삼키기---------------------------------------- \| 0 \| 1 \| 2 \| 3 \| 4 \| \| 1. 물 마시기---------------------------------------- \| 0 \| 1 \| 2 \| 3 \| 4 \| \| 1. 밥 먹기------------------------------------------- \| 0 \| 1 \| 2 \| 3 \| 4 \| \| 1. 말하기------------------------------------------- \| 0 \| 1 \| 2 \| 3 \| 4 \| \| 1. 수면---------------------------------------------- \| 0 \| 1 \| 2 \| 3 \| 4 \|   Q4. 지난 하루 동안 환자분의 전체적인 구강과 목의 통증을 점수로 표현한다면 다음 중 몇 점에 해당합니까?  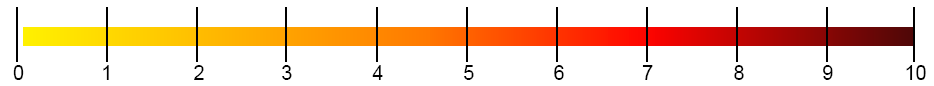  최고 통증   |
| --- | --- | --- | --- | --- | --- | --- | --- | --- | --- | --- | --- | --- | --- | --- | --- | --- | --- | --- | --- | --- | --- | --- | --- | --- | --- | --- | --- | --- | --- | --- | --- | --- | --- | --- | --- | --- | --- | --- | --- | --- | --- | --- | --- | --- | --- | --- | --- | --- | --- | --- | --- |

Reference) Patrick JS, Christos E, William IB, et al. Palifermin Reduces Patient-Reported Mouth and Throat

Soreness and Improves Patient Functioning in the Hematopoietic Stem-Cell Transplantation Setting. J Clin Oncol 2006; 24:5186-5193.

별첨6. ECOG Performance Status

| Grade | Status |
| --- | --- |
| 0 | 충분한 활동, 병을 앓기 이전의 모든 일상 생활을 제한 없이 수행할 수 있다. |
| 1 | 육체적으로 격렬한 활동에는 제한이 있으나, 보행 및 가볍게 할 수 있거나 앉아서 하는 업무(예를들어 가벼운 가사일, 회사일)은 수행할 수 있다. |
| 2 | 보행 및 자가 간호는 수행할 수 있으나, 직업적 활동은 수행할 수 없다. 깨어있는 시간의 50% 이상은 병상을 떠나 활동한다. |
| 3 | 제한된 자가 간호만을 수행할 수 있으며, 깨어있는 시간의 50% 이상을 침상 또는 의자에서 보낸다. |
| 4 | 완전한 불구, 어떠한 자가 간호도 수행할 수 없다. 완전히 침상 또는 의자에서만 보낸다. |
| 5 | 사망 |

Reference) Oken, MM, Creech RH, Tormey DC, et al. Toxicity And Response Criteria Of The Eastern Cooperative Oncology Group. Am J Clin Oncol 5:649-655, 1982.

별첨7. 임상시험약과 부작용과의 인과관계

| **Qustion** | **Yes** | **No** | **Unkown** | **Score** |
| --- | --- | --- | --- | --- |
| 1. 이 이상반응에 대해 약물과 관련하여 이전에 명확한 보고(previous conclusive reports)가 있었는가? | + 1 | 0 | 0 |  |
| 1. 의심 약물의 투여 후 이상반응이 발생하였는가? | + 2 | - 1 | 0 |  |
| 1. 투여 중단 또는 특정 길항제(specific antagonists)를 투여하였을 때 이상반응이 개선되었는가? | + 1 | 0 | 0 |  |
| 1. 재투여를 하였을 때 이전 이상반응과 동일한 이상반응이 발생하였는가? | +2 | - 1 | 0 |  |
| 1. 그 이상 반응에 영향을 끼칠 수 있는 다른 원인(alternative causes)이 존재하는가? | - 1 | + 2 | 0 |  |
| 1. 위약(placebo)을 투여하였을 경우에도 같은 반응이 발생하였는가? | - 1 | + 1 | 0 |  |
| 1. 환자의 체액(any body fluid)에서 의심 약물이 독성농도(toxic concentrations)로 관찰되었는가? | + 1 | 0 | 0 |  |
| 1. 약물의 양과 이상반응의 중증도 간에 비례관례가 있었는가? | + 1 | 0 | 0 |  |
| 1. 환자가 이전에 동일 또는 유사약물에 대해 유사한 반응을 보였는가? | + 1 | 0 | 0 |  |
| **Total score** | | | |  |

| **Total score** | **Indication** |
| --- | --- |
| > 9 | Definitely ADR |
| 5 ~ 8 | Probably ADR |
| 1 ~ 4 | Possibly ADR |
| 0 | doubtful |

Reference) Naranjo CA, Busto U, Sellers EM, et al. "A method for estimating the probability of adverse drug reactions". Clin. Pharmacol. Ther. 1981;30(2):239–45.

별첨8. 중대한 이상반응 보고서

**중대한 이상반응 보고서**

| 연구명 | 악성 혈액질환 환자에서 고용량 항암화학요법에 의해 발생하는 구내염에 대한 재조합 인간상피세포성장인자의 무작위 2상 임상 연구 | | | |
| --- | --- | --- | --- | --- |
| 연구자 | 책임연구자 | 윤 성 수 | | |
| 피험자 정보 | 피험자 번호 |  | | |
|  | 생년월일 |  | 성별 | □ 남 □ 여 |
|  | 진단명 |  | | |
| 임상시험약 | 임상시험약 번호 |  | | |
|  | 투약 기간 |  | | |
| 이상반응 | 이상반응명 |  | | |
|  | 발현일 및 소실일 |  | | |
|  | 시험약 관련 조치 |  | | |
|  | 치료 |  | | |
|  | 결과 |  | | |
| 임상시험약과의 인과관계 | □ 명확히 관련 있음 □ 관련이 있음 □ 관련가능성이 있음  □ 관련이 없음 □ 명확히 관련이 없음 □ 불명 | | | |
| 연구자 견해  (comment) |  | | | |
| 위와 같이 이상반응을 보고합니다.  보고일: 200 년 월 일  책임연구자: (인) | | | | |

별첨9. 증례기록지

**증례기록지**

**CASE REPORT FORM**

| **악성혈액질환 환자에서 고용량 항암화학요법에 의해 발생하는 구내염에 대한 재조합 인간상피세포성장인자의**  **무작위 2상 임상 연구**  **Randomized Phase II Study of Recombinant Human Epidermal Growth Factor (rhEGF) on Oral Mucositis Induced by Intensive Chemotherapy for Hematologic Malignancies** |
| --- |

**피험자 번호:**

**피험자이니셜:**

**책임연구자:**

| **선정 기준** | | | |
| --- | --- | --- | --- |
| **선정기준에 하나 이상의 ‘아니오’가 있을 경우, 본 연구에서 제외시킨다.** | | 예 | 아니오 |
| 1. 만 18세 이상의 성인 | | □ | □ |
| 1. 악성혈액질환이 확진 된 자 | | □ | □ |
| 1. 고용량 항암화학요법을 예정하고 있는 입원 환자 | | □ | □ |
| 1. ECOG performance status 0-2 | | □ | □ |
| 1. 본 연구에 참여하고자 서면 동의 한 자 | | □ | □ |
| **제외 기준** | | | |
| **제외기준에 하나 이상의 ‘예’가 있을 경우, 본 연구분석 대상에서 제외시킨다.** | 예 | | 아니오 |
| 1. 본 약제 또는 이와 비슷한 계열의 약제에 과민성을 갖는 자 | □ | | □ |
| 1. 항암화학요법 이전에 구강 내 궤양 또는 단순 포진이 있는 자. 즉, 중증도 평가도구- Common Terminology Criteria for Adverse Events v3.0에서 grade 0 이 아닌 경우 | □ | | □ |
| 1. 중증의 치과 질환이 있는 자 | □ | | □ |
| 1. 연구 참여 전 3주 이내에 항암화학용법 또는 방사선 치료를 받은 자 | □ | | □ |
| 1. 연구 참여 전 3주 이내에 외과적 수술을 받은 자 | □ | | □ |
| 1. 본 연구에 영향을 줄 수 있는 임상시험에 본 연구 참여 전 4주 이내에 참여하였거나 현재 참여 중인 자 | □ | | □ |
| 1. 악성혈액질환 외에 연구에 영향을 미칠 만큼의 중증 질환을 동반하고 있는 자 | □ | | □ |
| 1. 중증 정신 질환자 또는 약물 및 알코올 중독 환자 | □ | | □ |
| 1. 임신 중이거나 수유중인 여성 | □ | | □ |
| 1. 기타, 연구자에 의해 본 연구에 수행이 곤란하다고 판단되는 자 | □ | | □ |
| 1. 환자가 연구에 동의하지 않는 경우 | □ | | □ |

**상기 항목들을 종합하면, 환자의 임상연구 참여가 적합합니까?**

  예 □        아니오 □

**환자 동의한 날짜:**          년    월     일

**임상연구 책임연구자**

| 1. **Patient Essential Information** | | | | | | | | | | | | | | | | | | | | | | | | | | | | | | | | | | | |  |
| --- | --- | --- | --- | --- | --- | --- | --- | --- | --- | --- | --- | --- | --- | --- | --- | --- | --- | --- | --- | --- | --- | --- | --- | --- | --- | --- | --- | --- | --- | --- | --- | --- | --- | --- | --- | --- |
| **Patient’s name (initial)** | | | | | | | |  | | | | | | | | | | | | **Patient medical ID** | | | | | | | | |  | | | | | | |  |
| **Birthday (age)** | | | | | | | | 년 월 일 **( )** | | | | | | | | | | | | **Gender** | | | | | | | | | □ M □ F | | | | | | |  |
| **Diagnosis** | | | | | | | | □ AML □ ALL □ CML □ CLL □ MM  □ NHL □ AA □ ( ) | | | | | | | | | | | | | | | | | | | | | | | | | | | |  |
| **Admission day** | | | | | | | | 년 월 일 | | | | | | | | | | | | **Transplantation day** | | | | | | | | | 년 월 일 | | | | | | |  |
| **SCT type** | | | | | | | | □ AlloSCT ( □ sibling □ unrelated ) □ AutoSCT | | | | | | | | | | | | | | | | | | | | | | | | | | | |  |
| **Past Medical History** | | | | | | | | □ Hypertension ( / Meds: )  □ Renal disorder ( / Meds: )  □ Hepatic disorder ( / Meds: )  □ Diabetes ( / Meds: )  □ Allergies ( )  □ ( / Meds: ) | | | | | | | | | | | | | | | | | | | | | | | | | | | |  |
| **High-dose chemotherapy regimen** | | | | | | | | Name | | | | | | | | | | Dose | | | | | | | | Administration days | | | | | | | | | |  |
|  |  |  |  |  |  |  |  |  | | | | | | | | | |  | | | | | | | |  | | | | | | | | | |  |
|  |  |  |  |  |  |  |  |  | | | | | | | | | |  | | | | | | | |  | | | | | | | | | |  |
|  |  |  |  |  |  |  |  |  | | | | | | | | | |  | | | | | | | |  | | | | | | | | | |  |
|  |  |  |  |  |  |  |  |  | | | | | | | | | |  | | | | | | | |  | | | | | | | | | |  |
|  |  |  |  |  |  |  |  |  | | | | | | | | | |  | | | | | | | |  | | | | | | | | | |  |
|  |  |  |  |  |  |  |  |  | | | | | | | | | |  | | | | | | | |  | | | | | | | | | |  |
|  |  |  |  |  |  |  |  |  | | | | | | | | | |  | | | | | | | |  | | | | | | | | | |  |
| **Concomitant Drugs**  **for SCT** | | | | | | | | Name | | | | | | | | | | Dose | | | | | | | | Start day | | | | | | | Duration | | |  |
|  |  |  |  |  |  |  |  | □ allopurinol | | | | | | | | | |  | | | | | | | |  | | | | | | |  | | |  |
|  |  |  |  |  |  |  |  | □ heparin | | | | | | | | | |  | | | | | | | |  | | | | | | |  | | |  |
|  |  |  |  |  |  |  |  | □ ciprofloxacin | | | | | | | | | |  | | | | | | | |  | | | | | | |  | | |  |
|  |  |  |  |  |  |  |  | □ fluconazole | | | | | | | | | |  | | | | | | | |  | | | | | | |  | | |  |
|  |  |  |  |  |  |  |  | □ sulfamethoxazole/  trimethoprim | | | | | | | | | |  | | | | | | | |  | | | | | | |  | | |  |
|  |  |  |  |  |  |  |  | □ IVIG | | | | | | | | | |  | | | | | | | |  | | | | | | |  | | |  |
|  |  |  |  |  |  |  |  | □ filgrastim (parenteral) | | | | | | | | | |  | | | | | | | |  | | | | | | |  | | |  |
|  |  |  |  |  |  |  |  | □ lenograstim (parenteral) | | | | | | | | | |  | | | | | | | |  | | | | | | |  | | |  |
|  |  |  |  |  |  |  |  | □ betadine gargle | | | | | | | | | |  | | | | | | | |  | | | | | | |  | | |  |
|  |  |  |  |  |  |  |  | □ nystatin gargle | | | | | | | | | |  | | | | | | | |  | | | | | | |  | | |  |
|  |  |  |  |  |  |  |  | □ CSA | | | | | | | | | |  | | | | | | | |  | | | | | | |  | | |  |
|  |  |  |  |  |  |  |  | □ ( ) | | | | | | | | | |  | | | | | | | |  | | | | | | |  | | |  |
| 1. **Baseline Characteristics 평가일: 년 월 일** | | | | | | | | | | | | | | | | | | | | | | | | | | | | | | | | | | | |  |
| **ECOG grade** | | | | | | | | | | 0 □ 1 □ 2 □ 3 □ 4 □ | | | | | | | | | | | | | | | | | | | | | | | | | |  |
| **Clinical data** | | | | | | | | | | Height | | | | | | | | | | | | | | | cm | | | | | | | | | | |  |
|  |  |  |  |  |  |  |  |  |  | Body weight | | | | | | | | | | | | | | | Kg | | | | | | | | | | |  |
|  |  |  |  |  |  |  |  |  |  | Blood pressure | | | | | | | | | | | | | | | / mmHg | | | | | | | | | | |  |
| **Lab data** | | | | | | | | | | WBC count | | | | | | | | | | | | | | | /㎣ | | | | | | | | | | |  |
|  |  |  |  |  |  |  |  |  |  | Hemoglobin | | | | | | | | | | | | | | | g/dL | | | | | | | | | | |  |
|  |  |  |  |  |  |  |  |  |  | Platelet count | | | | | | | | | | | | | | | 10^3^/㎣ | | | | | | | | | | |  |
|  |  |  |  |  |  |  |  |  |  | ANC | | | | | | | | | | | | | | | /㎣ | | | | | | | | | | |  |
|  |  |  |  |  |  |  |  |  |  | BUN/ Serum creatinine | | | | | | | | | | | | | | | / mg/dL | | | | | | | | | | |  |
|  |  |  |  |  |  |  |  |  |  | sGOT/ sGPT | | | | | | | | | | | | | | | / IU/L | | | | | | | | | | |  |
|  |  |  |  |  |  |  |  |  |  | Total Bilirubin/ Direct Bilirubin | | | | | | | | | | | | | | | / mg/dL | | | | | | | | | | |  |
| **Oral status** | | | | | | | | | | NCI CTCAE v3.0 grade ^2)^ | | | | | | | | | | | | | | | 0 □ 1 □ 2 □ 3 □ 4 □ | | | | | | | | | | |  |
|  |  |  |  |  |  |  |  |  |  | WHO Oral mucositis scoring ^3)^ | | | | | | | | | | | | | | | 0 □ 1 □ 2 □ 3 □ 4 □ | | | | | | | | | | |  |
| 1. **Efficacy Evaluation** | | | | | | | | | | | | | | | | | | | | | | | | | | | | | | | | | | | |  |
| DAY | | | | | | | **D-8** | | | | **D-7** | | | | **D-6** | | | | **D-5** | | | | **D-4** | | | | **D-3** | | | | **D-2** | | | **D-1** | |  |
| Date | | | | | | |  | | | |  | | | |  | | | |  | | | |  | | | |  | | | |  | | |  | |  |
| NCI CTCAE V3.0 grade  (grade 0~4) | | | | | | |  | | | |  | | | |  | | | |  | | | |  | | | |  | | | |  | | |  | |  |
| WHO oral mucositis score  (grade 0~4) | | | | | | |  | | | |  | | | |  | | | |  | | | |  | | | |  | | | |  | | |  | |  |
| OMDQ  Score  (Q1~Q4) | | Q1 (0~10) | | | | |  | | | |  | | | |  | | | |  | | | |  | | | |  | | | |  | | |  | |  |
|  |  | Q2 (0~4) | | | | |  | | | |  | | | |  | | | |  | | | |  | | | |  | | | |  | | |  | |  |
|  |  | Q3  (0~4) | | a | | |  | | | |  | | | |  | | | |  | | | |  | | | |  | | | |  | | |  | |  |
|  |  |  |  | b | | |  | | | |  | | | |  | | | |  | | | |  | | | |  | | | |  | | |  | |  |
|  |  |  |  | c | | |  | | | |  | | | |  | | | |  | | | |  | | | |  | | | |  | | |  | |  |
|  |  |  |  | d | | |  | | | |  | | | |  | | | |  | | | |  | | | |  | | | |  | | |  | |  |
|  |  |  |  | e | | |  | | | |  | | | |  | | | |  | | | |  | | | |  | | | |  | | |  | |  |
|  |  | Q4 (0~10) | | | | |  | | | |  | | | |  | | | |  | | | |  | | | |  | | | |  | | |  | |  |
| Use of Opioid analgesic | | | | | | | □Y □N | | | | □Y □N | | | | □Y □N | | | | □Y □N | | | | □Y □N | | | | □Y □N | | | | □Y □N | | | □Y □N | |  |
| Use of TPN | | | | | | | □Y □N | | | | □Y □N | | | | □Y □N | | | | □Y □N | | | | □Y □N | | | | □Y □N | | | | □Y □N | | | □Y □N | |  |
| **III. Efficacy Evaluation (continued)** | | | | | | | | | | | | | | | | | | | | | | | | | | | | | | | | | | | |  |
| **DAY** | | | | | | | **D 0** | | | | **D 1** | | | | **D 2** | | | | **D 3** | | | | **D 4** | | | | **D 5** | | | | **D 6** | | | **D 7** | |  |
| Date | | | | | | |  | | | |  | | | |  | | | |  | | | |  | | | |  | | | |  | | |  | |  |
| NCI CTCAE V3.0 grade  (grade 0~4) | | | | | | |  | | | |  | | | |  | | | |  | | | |  | | | |  | | | |  | | |  | |  |
| WHO oral mucositis score  (grade 0~4) | | | | | | |  | | | |  | | | |  | | | |  | | | |  | | | |  | | | |  | | |  | |  |
| OMDQ  Score  (Q1~Q4) | | Q1 (0~10) | | | | |  | | | |  | | | |  | | | |  | | | |  | | | |  | | | |  | | |  | |  |
|  |  | Q2 (0~4) | | | | |  | | | |  | | | |  | | | |  | | | |  | | | |  | | | |  | | |  | |  |
|  |  | Q3  (0~4) | | a | | |  | | | |  | | | |  | | | |  | | | |  | | | |  | | | |  | | |  | |  |
|  |  |  |  | b | | |  | | | |  | | | |  | | | |  | | | |  | | | |  | | | |  | | |  | |  |
|  |  |  |  | c | | |  | | | |  | | | |  | | | |  | | | |  | | | |  | | | |  | | |  | |  |
|  |  |  |  | d | | |  | | | |  | | | |  | | | |  | | | |  | | | |  | | | |  | | |  | |  |
|  |  |  |  | e | | |  | | | |  | | | |  | | | |  | | | |  | | | |  | | | |  | | |  | |  |
|  |  | Q4 (0~10) | | | | |  | | | |  | | | |  | | | |  | | | |  | | | |  | | | |  | | |  | |  |
| Use of Opioid analgesic | | | | | | | □Y □N | | | | □Y □N | | | | □Y □N | | | | □Y □N | | | | □Y □N | | | | □Y □N | | | | □Y □N | | | □Y □N | |  |
| Use of TPN | | | | | | | □Y □N | | | | □Y □N | | | | □Y □N | | | | □Y □N | | | | □Y □N | | | | □Y □N | | | | □Y □N | | | □Y □N | |  |
| **DAY** | | | | | | | **D 8** | | | | **D 9** | | | | **D 10** | | | | **D 11** | | | | **D 12** | | | | **D 13** | | | | **D 14** | | | **D 15** | |  |
| Date | | | | | | |  | | | |  | | | |  | | | |  | | | |  | | | |  | | | |  | | |  | |  |
| NCI CTCAE V3.0 grade  (grade 0~4) | | | | | | |  | | | |  | | | |  | | | |  | | | |  | | | |  | | | |  | | |  | |  |
| WHO oral mucositis score  (grade 0~4) | | | | | | |  | | | |  | | | |  | | | |  | | | |  | | | |  | | | |  | | |  | |  |
| OMDQ  Score  (Q1~Q4) | | Q1 (0~10) | | | | |  | | | |  | | | |  | | | |  | | | |  | | | |  | | | |  | | |  | |  |
|  |  | Q2 (0~4) | | | | |  | | | |  | | | |  | | | |  | | | |  | | | |  | | | |  | | |  | |  |
|  |  | Q3  (0~4) | | a | | |  | | | |  | | | |  | | | |  | | | |  | | | |  | | | |  | | |  | |  |
|  |  |  |  | b | | |  | | | |  | | | |  | | | |  | | | |  | | | |  | | | |  | | |  | |  |
|  |  |  |  | c | | |  | | | |  | | | |  | | | |  | | | |  | | | |  | | | |  | | |  | |  |
|  |  |  |  | d | | |  | | | |  | | | |  | | | |  | | | |  | | | |  | | | |  | | |  | |  |
|  |  |  |  | e | | |  | | | |  | | | |  | | | |  | | | |  | | | |  | | | |  | | |  | |  |
|  |  | Q4 (0~10) | | | | |  | | | |  | | | |  | | | |  | | | |  | | | |  | | | |  | | |  | |  |
| Use of Opioid analgesic | | | | | | | □Y □N | | | | □Y □N | | | | □Y □N | | | | □Y □N | | | | □Y □N | | | | □Y □N | | | | □Y □N | | | □Y □N | |  |
| Use of TPN | | | | | | | □Y □N | | | | □Y □N | | | | □Y □N | | | | □Y □N | | | | □Y □N | | | | □Y □N | | | | □Y □N | | | □Y □N | |  |
| **III. Efficacy Evaluation (continued)** | | | | | | | | | | | | | | | | | | | | | | | | | | | | | | | | | | | |  |
| **DAY** | | | | | | | **D 16** | | | | **D 17** | | | | **D 18** | | | | **D 19** | | | | **D 20** | | | | **D 21** | | | | **D 22** | | | **D 23** | |  |
| Date | | | | | | |  | | | |  | | | |  | | | |  | | | |  | | | |  | | | |  | | |  | |  |
| NCI CTCAE V3.0 grade  (grade 0~4) | | | | | | |  | | | |  | | | |  | | | |  | | | |  | | | |  | | | |  | | |  | |  |
| WHO oral mucositis score  (grade 0~4) | | | | | | |  | | | |  | | | |  | | | |  | | | |  | | | |  | | | |  | | |  | |  |
| OMDQ  Score  (Q1~Q4) | | Q1 (0~10) | | | | |  | | | |  | | | |  | | | |  | | | |  | | | |  | | | |  | | |  | |  |
|  |  | Q2 (0~4) | | | | |  | | | |  | | | |  | | | |  | | | |  | | | |  | | | |  | | |  | |  |
|  |  | Q3  (0~4) | | a | | |  | | | |  | | | |  | | | |  | | | |  | | | |  | | | |  | | |  | |  |
|  |  |  |  | b | | |  | | | |  | | | |  | | | |  | | | |  | | | |  | | | |  | | |  | |  |
|  |  |  |  | c | | |  | | | |  | | | |  | | | |  | | | |  | | | |  | | | |  | | |  | |  |
|  |  |  |  | d | | |  | | | |  | | | |  | | | |  | | | |  | | | |  | | | |  | | |  | |  |
|  |  |  |  | e | | |  | | | |  | | | |  | | | |  | | | |  | | | |  | | | |  | | |  | |  |
|  |  | Q4 (0~10) | | | | |  | | | |  | | | |  | | | |  | | | |  | | | |  | | | |  | | |  | |  |
| Use of Opioid analgesic | | | | | | | □Y □N | | | | □Y □N | | | | □Y □N | | | | □Y □N | | | | □Y □N | | | | □Y □N | | | | □Y □N | | | □Y □N | |  |
| Use of TPN | | | | | | | □Y □N | | | | □Y □N | | | | □Y □N | | | | □Y □N | | | | □Y □N | | | | □Y □N | | | | □Y □N | | | □Y □N | |  |
| **DAY** | | | | | | | **D 24** | | | | **D 25** | | | | **D 26** | | | | **D 27** | | | | **D 28** | | | | **D 29** | | | | **D 30** | | | **D 31** | |  |
| Date | | | | | | |  | | | |  | | | |  | | | |  | | | |  | | | |  | | | |  | | |  | |  |
| NCI CTCAE V3.0 grade  (grade 0~4) | | | | | | |  | | | |  | | | |  | | | |  | | | |  | | | |  | | | |  | | |  | |  |
| WHO oral mucositis score  (grade 0~4) | | | | | | |  | | | |  | | | |  | | | |  | | | |  | | | |  | | | |  | | |  | |  |
| OMDQ  Score  (Q1~Q4) | | Q1 (0~10) | | | | |  | | | |  | | | |  | | | |  | | | |  | | | |  | | | |  | | |  | |  |
|  |  | Q2 (0~4) | | | | |  | | | |  | | | |  | | | |  | | | |  | | | |  | | | |  | | |  | |  |
|  |  | Q3  (0~4) | | a | | |  | | | |  | | | |  | | | |  | | | |  | | | |  | | | |  | | |  | |  |
|  |  |  |  | b | | |  | | | |  | | | |  | | | |  | | | |  | | | |  | | | |  | | |  | |  |
|  |  |  |  | c | | |  | | | |  | | | |  | | | |  | | | |  | | | |  | | | |  | | |  | |  |
|  |  |  |  | d | | |  | | | |  | | | |  | | | |  | | | |  | | | |  | | | |  | | |  | |  |
|  |  |  |  | e | | |  | | | |  | | | |  | | | |  | | | |  | | | |  | | | |  | | |  | |  |
|  |  | Q4 (0~10) | | | | |  | | | |  | | | |  | | | |  | | | |  | | | |  | | | |  | | |  | |  |
| Use of Opioid analgesic | | | | | | | □Y □N | | | | □Y □N | | | | □Y □N | | | | □Y □N | | | | □Y □N | | | | □Y □N | | | | □Y □N | | | □Y □N | |  |
| Use of TPN | | | | | | | □Y □N | | | | □Y □N | | | | □Y □N | | | | □Y □N | | | | □Y □N | | | | □Y □N | | | | □Y □N | | | □Y □N | |  |
| 1. **Safety Evaluation** | | | | | | | | | | | | | | | | | | | | | | | | | | | | | | | | | | | | |
| **Adverse Reactions**  **of**  **Study Drug** | | | | **DAY** | | | | | | **D-8** | | | **D-7** | | | | **D-6** | | | | | **D-5** | | | **D-4** | | | | **D-3** | | | | **D-2** | | | **D-1** |
|  |  |  |  | Date | | | | | |  | | |  | | | |  | | | | |  | | |  | | | |  | | | |  | | |  |
|  |  |  |  | Type | | | | | |  | | |  | | | |  | | | | |  | | |  | | | |  | | | |  | | |  |
|  |  |  |  | Severity  CTCAE v3.0 | | | | | | □ grade 1  □ grade 2  □ grade 3  □ grade 4  □ grade 5 | | | □ grade 1  □ grade 2  □ grade 3  □ grade 4  □ grade 5 | | | | □ grade 1  □ grade 2  □ grade 3  □ grade 4  □ grade 5 | | | | | □ grade 1  □ grade 2  □ grade 3  □ grade 4  □ grade 5 | | | □ grade 1  □ grade 2  □ grade 3  □ grade 4  □ grade 5 | | | | □ grade 1  □ grade 2  □ grade 3  □ grade 4  □ grade 5 | | | | □ grade 1  □ grade 2  □ grade 3  □ grade 4  □ grade 5 | | | □ grade 1  □ grade 2  □ grade 3  □ grade 4  □ grade 5 |
|  |  |  |  | Relationship with  EGF sol.  Naranjo scale | | | | | | □definite  □probable  □possible  □doubtful | | | □definite  □probable  □possible  □doubtful | | | | □definite  □probable  □possible  □doubtful | | | | | □definite  □probable  □possible  □doubtful | | | □definite  □probable  □possible  □doubtful | | | | □definite  □probable  □possible  □doubtful | | | | □definite  □probable  □possible  □doubtful | | | □definite  □probable  □possible  □doubtful |
|  |  |  |  | EGF continuation | | | | | | □continued  □D/C | | | □continued  □D/C | | | | continued  □D/C | | | | | □continued  □D/C | | | □continued  □D/C | | | | □continued  □D/C | | | | □continued  □D/C | | | □continued  □D/C |
|  |  |  |  | Treatment | | | | | |  | | |  | | | |  | | | | |  | | |  | | | |  | | | |  | | |  |
|  |  |  |  | **DAY** | | | | | | **D 0** | | | **D 1** | | | | **D 2** | | | | | **D 3** | | | **D 4** | | | | **D 5** | | | | **D 6** | | | **D 7** |
|  |  |  |  | Date | | | | | |  | | |  | | | |  | | | | |  | | |  | | | |  | | | |  | | |  |
|  |  |  |  | Type | | | | | |  | | |  | | | |  | | | | |  | | |  | | | |  | | | |  | | |  |
|  |  |  |  | Severity  CTCAE v3.0 | | | | | | □ grade 1  □ grade 2  □ grade 3  □ grade 4  □ grade 5 | | | □ grade 1  □ grade 2  □ grade 3  □ grade 4  □ grade 5 | | | | □ grade 1  □ grade 2  □ grade 3  □ grade 4  □ grade 5 | | | | | □ grade 1  □ grade 2  □ grade 3  □ grade 4  □ grade 5 | | | □ grade 1  □ grade 2  □ grade 3  □ grade 4  □ grade 5 | | | | □ grade 1  □ grade 2  □ grade 3  □ grade 4  □ grade 5 | | | | □ grade 1  □ grade 2  □ grade 3  □ grade 4  □ grade 5 | | | □ grade 1  □ grade 2  □ grade 3  □ grade 4  □ grade 5 |
|  |  |  |  | Relationship with  EGF sol.  Naranjo scale | | | | | | □definite  □probable  □possible  □doubtful | | | □definite  □probable  □possible  □doubtful | | | | □definite  □probable  □possible  □doubtful | | | | | □definite  □probable  □possible  □doubtful | | | □definite  □probable  □possible  □doubtful | | | | □definite  □probable  □possible  □doubtful | | | | □definite  □probable  □possible  □doubtful | | | □definite  □probable  □possible  □doubtful |
|  |  |  |  | EGF continuation | | | | | | □continued  □D/C | | | □continued  □D/C | | | | □continued  □D/C | | | | | □continued  □D/C | | | □continued  □D/C | | | | □continued  □D/C | | | | □continued  □D/C | | | □continued  □D/C |
|  |  |  |  | Treatment | | | | | |  | | |  | | | |  | | | | |  | | |  | | | |  | | | |  | | |  |
| **IV. Safety Evaluation (continued)** | | | | | | | | | | | | | | | | | | | | | | | | | | | | | | | | | | | | |
| **Adverse Reactions**  **of**  **Study Drug** | | | | **DAY** | | | | | | **D 8** | | | **D 9** | | | | **D 10** | | | | | **D 11** | | | **D 12** | | | | **D 13** | | | | **D 14** | | | **D 15** |
|  |  |  |  | Date | | | | | |  | | |  | | | |  | | | | |  | | |  | | | |  | | | |  | | |  |
|  |  |  |  | Type | | | | | |  | | |  | | | |  | | | | |  | | |  | | | |  | | | |  | | |  |
|  |  |  |  | Severity  CTCAE v3.0 | | | | | | □ grade 1  □ grade 2  □ grade 3  □ grade 4  □ grade 5 | | | □ grade 1  □ grade 2  □ grade 3  □ grade 4  □ grade 5 | | | | □ grade 1  □ grade 2  □ grade 3  □ grade 4  □ grade 5 | | | | | □ grade 1  □ grade 2  □ grade 3  □ grade 4  □ grade 5 | | | □ grade 1  □ grade 2  □ grade 3  □ grade 4  □ grade 5 | | | | □ grade 1  □ grade 2  □ grade 3  □ grade 4  □ grade 5 | | | | □ grade 1  □ grade 2  □ grade 3  □ grade 4  □ grade 5 | | | □ grade 1  □ grade 2  □ grade 3  □ grade 4  □ grade 5 |
|  |  |  |  | Relationship with  EGF sol.  Naranjo scale | | | | | | □definite  □probable  □possible  □doubtful | | | □definite  □probable  □possible  □doubtful | | | | □definite  □probable  □possible  □doubtful | | | | | □definite  □probable  □possible  □doubtful | | | □definite  □probable  □possible  □doubtful | | | | □definite  □probable  □possible  □doubtful | | | | □definite  □probable  □possible  □doubtful | | | □definite  □probable  □possible  □doubtful |
|  |  |  |  | EGF continuation | | | | | | □continued  □D/C | | | □continued  □D/C | | | | continued  □D/C | | | | | □continued  □D/C | | | □continued  □D/C | | | | □continued  □D/C | | | | □continued  □D/C | | | □continued  □D/C |
|  |  |  |  | Treatment | | | | | |  | | |  | | | |  | | | | |  | | |  | | | |  | | | |  | | |  |
|  |  |  |  | **DAY** | | | | | | **D 16** | | | **D 17** | | | | **D 18** | | | | | **D 19** | | | **D 20** | | | | **D 21** | | | | **D 22** | | | **D 23** |
|  |  |  |  | Date | | | | | |  | | |  | | | |  | | | | |  | | |  | | | |  | | | |  | | |  |
|  |  |  |  | Type | | | | | |  | | |  | | | |  | | | | |  | | |  | | | |  | | | |  | | |  |
|  |  |  |  | Severity  CTCAE v3.0 | | | | | | □ grade 1  □ grade 2  □ grade 3  □ grade 4  □ grade 5 | | | □ grade 1  □ grade 2  □ grade 3  □ grade 4  □ grade 5 | | | | □ grade 1  □ grade 2  □ grade 3  □ grade 4  □ grade 5 | | | | | □ grade 1  □ grade 2  □ grade 3  □ grade 4  □ grade 5 | | | □ grade 1  □ grade 2  □ grade 3  □ grade 4  □ grade 5 | | | | □ grade 1  □ grade 2  □ grade 3  □ grade 4  □ grade 5 | | | | □ grade 1  □ grade 2  □ grade 3  □ grade 4  □ grade 5 | | | □ grade 1  □ grade 2  □ grade 3  □ grade 4  □ grade 5 |
|  |  |  |  | Relationship with  EGF sol.  Naranjo scale | | | | | | □definite  □probable  □possible  □doubtful | | | □definite  □probable  □possible  □doubtful | | | | □definite  □probable  □possible  □doubtful | | | | | □definite  □probable  □possible  □doubtful | | | □definite  □probable  □possible  □doubtful | | | | □definite  □probable  □possible  □doubtful | | | | □definite  □probable  □possible  □doubtful | | | □definite  □probable  □possible  □doubtful |
|  |  |  |  | EGF continuation | | | | | | □continued  □D/C | | | □continued  □D/C | | | | □continued  □D/C | | | | | □continued  □D/C | | | □continued  □D/C | | | | □continued  □D/C | | | | □continued  □D/C | | | □continued  □D/C |
|  |  |  |  | Treatment | | | | | |  | | |  | | | |  | | | | |  | | |  | | | |  | | | |  | | |  |
| **IV. Safety Evaluation (continued)** | | | | | | | | | | | | | | | | | | | | | | | | | | | | | | | | | | | | |
| **Adverse Reactions**  **of**  **Study Drug** | | | | **DAY** | | | | | | **D 24** | | | **D 25** | | | | **D 26** | | | | | **D 27** | | | **D 28** | | | | **D 29** | | | | **D 30** | | | **D 31** |
|  |  |  |  | Date | | | | | |  | | |  | | | |  | | | | |  | | |  | | | |  | | | |  | | |  |
|  |  |  |  | Type | | | | | |  | | |  | | | |  | | | | |  | | |  | | | |  | | | |  | | |  |
|  |  |  |  | Severity  CTCAE v3.0 | | | | | | □ grade 1  □ grade 2  □ grade 3  □ grade 4  □ grade 5 | | | □ grade 1  □ grade 2  □ grade 3  □ grade 4  □ grade 5 | | | | □ grade 1  □ grade 2  □ grade 3  □ grade 4  □ grade 5 | | | | | □ grade 1  □ grade 2  □ grade 3  □ grade 4  □ grade 5 | | | □ grade 1  □ grade 2  □ grade 3  □ grade 4  □ grade 5 | | | | □ grade 1  □ grade 2  □ grade 3  □ grade 4  □ grade 5 | | | | □ grade 1  □ grade 2  □ grade 3  □ grade 4  □ grade 5 | | | □ grade 1  □ grade 2  □ grade 3  □ grade 4  □ grade 5 |
|  |  |  |  | Relationship with  EGF sol.  Naranjo scale | | | | | | □definite  □probable  □possible  □doubtful | | | □definite  □probable  □possible  □doubtful | | | | □definite  □probable  □possible  □doubtful | | | | | □definite  □probable  □possible  □doubtful | | | □definite  □probable  □possible  □doubtful | | | | □definite  □probable  □possible  □doubtful | | | | □definite  □probable  □possible  □doubtful | | | □definite  □probable  □possible  □doubtful |
|  |  |  |  | EGF continuation | | | | | | □continued  □D/C | | | □continued  □D/C | | | | continued  □D/C | | | | | □continued  □D/C | | | □continued  □D/C | | | | □continued  □D/C | | | | □continued  □D/C | | | □continued  □D/C |
|  |  |  |  | Treatment | | | | | |  | | |  | | | |  | | | | |  | | |  | | | |  | | | |  | | |  |
| 1. **Compliance of Study Drug** | | | | | | | | | | | | | | | | | | | | | | | | | | | | | | | | | | | | |
| **Compliance of study drug** | | | | **DAY** | | | | | | **D -8** | | | **D -7** | | | | **D -6** | | | | | **D -5** | | | **D -4** | | | | **D -3** | | | | **D -2** | | | **D -1** |
|  |  |  |  | Date | | | | | |  | | |  | | | |  | | | | |  | | |  | | | |  | | | |  | | |  |
|  |  |  |  | Compliance (%) | | | | | |  | | |  | | | |  | | | | |  | | |  | | | |  | | | |  | | |  |
|  |  |  |  | **DAY** | | | | | | **D 0** | | | **D 1** | | | | **D 2** | | | | | **D 3** | | | **D 4** | | | | **D 5** | | | | **D 6** | | | **D 7** |
|  |  |  |  | Date | | | | | |  | | |  | | | |  | | | | |  | | |  | | | |  | | | |  | | |  |
|  |  |  |  | Compliance (%) | | | | | |  | | |  | | | |  | | | | |  | | |  | | | |  | | | |  | | |  |
|  |  |  |  | **DAY** | | | | | | **D 8** | | | **D 9** | | | | **D 10** | | | | | **D 11** | | | **D 12** | | | | **D 13** | | | | **D 14** | | | **D 15** |
|  |  |  |  | Date | | | | | |  | | |  | | | |  | | | | |  | | |  | | | |  | | | |  | | |  |
|  |  |  |  | Compliance (%) | | | | | |  | | |  | | | |  | | | | |  | | |  | | | |  | | | |  | | |  |
|  |  |  |  | **DAY** | | | | | | **D 16** | | | **D 17** | | | | **D 18** | | | | | **D 19** | | | **D 20** | | | | **D 21** | | | | **D 22** | | | **D 23** |
|  |  |  |  | Date | | | | | |  | | |  | | | |  | | | | |  | | |  | | | |  | | | |  | | |  |
|  |  |  |  | Compliance (%) | | | | | |  | | |  | | | |  | | | | |  | | |  | | | |  | | | |  | | |  |
| **V. Compliance of Study Drug (continued)** | | | | | | | | | | | | | | | | | | | | | | | | | | | | | | | | | | | | |
| **Compliance of study drug** | | | | **DAY** | | | | | | **D 24** | | | **D 25** | | | | **D 26** | | | | | **D 27** | | | **D 28** | | | | **D 29** | | | | **D 30** | | | **D 31** |
|  |  |  |  | Date | | | | | |  | | |  | | | |  | | | | |  | | |  | | | |  | | | |  | | |  |
|  |  |  |  | Compliance (%) | | | | | |  | | |  | | | |  | | | | |  | | |  | | | |  | | | |  | | |  |
| 1. **Infection after SCT** | | | | | | | | | | | | | | | | | | | | | | | | | | | | | | | | | | | | |
| Date | | Class | | | | | Site of Infection | | | | | | | Category of infection | | | | | | | | | Organism (if MDI) | | | | | | | | Empiric/Treatment | | | | | |
|  | | □MDI  □CDI  □UF | | | | | □ blood  □ CNS  □ lung  □ sputum  □ abdomen  □ GU tract  □ skin & soft tissue  □ C-line  □ ( ) | | | | | | | □ bacterial  □ fungal  □ TB  □ viral  □ PCP  □ mixed  □ other | | | | | | | | |  | | | | | | | | **Empiric** | | | | | |
|  |  |  |  |  |  |  |  |  |  |  |  |  |  |  |  |  |  |  |  |  |  |  |  |  |  |  |  |  |  |  |  | | | | | |
|  |  |  |  |  |  |  |  |  |  |  |  |  |  |  |  |  |  |  |  |  |  |  |  |  |  |  |  |  |  |  |  | | | | | |
|  |  |  |  |  |  |  |  |  |  |  |  |  |  |  |  |  |  |  |  |  |  |  |  |  |  |  |  |  |  |  |  | | | | | |
|  |  |  |  |  |  |  |  |  |  |  |  |  |  |  |  |  |  |  |  |  |  |  |  |  |  |  |  |  |  |  | **Treatment** | | | | | |
|  |  |  |  |  |  |  |  |  |  |  |  |  |  |  |  |  |  |  |  |  |  |  |  |  |  |  |  |  |  |  |  | | | | | |
|  |  |  |  |  |  |  |  |  |  |  |  |  |  |  |  |  |  |  |  |  |  |  |  |  |  |  |  |  |  |  |  | | | | | |
|  |  |  |  |  |  |  |  |  |  |  |  |  |  |  |  |  |  |  |  |  |  |  |  |  |  |  |  |  |  |  |  | | | | | |
| **NOTE** | | | | | | | | | | | | | | | | | | | | | | | | | | | | | | | | | | | | |
|  | | □MDI  □CDI  □UF | | | | | □ blood  □ CNS  □ lung  □ sputum  □ abdomen  □ GU tract  □ skin & soft tissue  □ C-line  □ ( ) | | | | | | | □ bacterial  □ fungal  □ TB  □ viral  □ PCP  □ mixed  □ other | | | | | | | | |  | | | | | | | | **Empiric** | | | | | |
|  |  |  |  |  |  |  |  |  |  |  |  |  |  |  |  |  |  |  |  |  |  |  |  |  |  |  |  |  |  |  |  | | | | | |
|  |  |  |  |  |  |  |  |  |  |  |  |  |  |  |  |  |  |  |  |  |  |  |  |  |  |  |  |  |  |  |  | | | | | |
|  |  |  |  |  |  |  |  |  |  |  |  |  |  |  |  |  |  |  |  |  |  |  |  |  |  |  |  |  |  |  |  | | | | | |
|  |  |  |  |  |  |  |  |  |  |  |  |  |  |  |  |  |  |  |  |  |  |  |  |  |  |  |  |  |  |  | **Treatment** | | | | | |
|  |  |  |  |  |  |  |  |  |  |  |  |  |  |  |  |  |  |  |  |  |  |  |  |  |  |  |  |  |  |  |  | | | | | |
|  |  |  |  |  |  |  |  |  |  |  |  |  |  |  |  |  |  |  |  |  |  |  |  |  |  |  |  |  |  |  |  | | | | | |
|  |  |  |  |  |  |  |  |  |  |  |  |  |  |  |  |  |  |  |  |  |  |  |  |  |  |  |  |  |  |  |  | | | | | |
| **NOTE** | | | | | | | | | | | | | | | | | | | | | | | | | | | | | | | | | | | | |
|  | | □MDI  □CDI  □UF | | | | | □ blood  □ CNS  □ lung  □ sputum  □ abdomen  □ GU tract  □ skin & soft tissue  □ C-line  □ ( ) | | | | | | | □ bacterial  □ fungal  □ TB  □ viral  □ PCP  □ mixed  □ other | | | | | | | | |  | | | | | | | | **Empiric** | | | | | |
|  |  |  |  |  |  |  |  |  |  |  |  |  |  |  |  |  |  |  |  |  |  |  |  |  |  |  |  |  |  |  |  | | | | | |
|  |  |  |  |  |  |  |  |  |  |  |  |  |  |  |  |  |  |  |  |  |  |  |  |  |  |  |  |  |  |  |  | | | | | |
|  |  |  |  |  |  |  |  |  |  |  |  |  |  |  |  |  |  |  |  |  |  |  |  |  |  |  |  |  |  |  |  | | | | | |
|  |  |  |  |  |  |  |  |  |  |  |  |  |  |  |  |  |  |  |  |  |  |  |  |  |  |  |  |  |  |  | **Treatment** | | | | | |
|  |  |  |  |  |  |  |  |  |  |  |  |  |  |  |  |  |  |  |  |  |  |  |  |  |  |  |  |  |  |  |  | | | | | |
|  |  |  |  |  |  |  |  |  |  |  |  |  |  |  |  |  |  |  |  |  |  |  |  |  |  |  |  |  |  |  |  | | | | | |
|  |  |  |  |  |  |  |  |  |  |  |  |  |  |  |  |  |  |  |  |  |  |  |  |  |  |  |  |  |  |  |  | | | | | |
| **NOTE** | | | | | | | | | | | | | | | | | | | | | | | | | | | | | | | | | | | | |
| 1. **Acute GVHD after SCT** | | | | | | | | | | | | | | | | | | | | | | | | | | | | | | | | | | | | |
| Diagnosis date | | | | | | Site of GVHD | | | | | | | | | Grade | | | | | | | | | Treatment | | | | | | | | | | | | |
|  | | | | | | □ liver  □ skin  □ GI tract  □ systemic  □ ( ) | | | | | | | | | □ grade 1  □ grade 2  □ grade 3  □ grade 4 | | | | | | | | |  | | | | | | | | | | | | |
|  |  |  |  |  |  |  |  |  |  |  |  |  |  |  |  |  |  |  |  |  |  |  |  |  | | | | | | | | | | | | |
|  |  |  |  |  |  |  |  |  |  |  |  |  |  |  |  |  |  |  |  |  |  |  |  |  | | | | | | | | | | | | |
|  |  |  |  |  |  |  |  |  |  |  |  |  |  |  |  |  |  |  |  |  |  |  |  |  | | | | | | | | | | | | |
|  |  |  |  |  |  |  |  |  |  |  |  |  |  |  |  |  |  |  |  |  |  |  |  |  | | | | | | | | | | | | |
|  | | | | | | □ liver  □ skin  □ GI tract  □ systemic  □ ( ) | | | | | | | | | □ grade 1  □ grade 2  □ grade 3  □ grade 4 | | | | | | | | |  | | | | | | | | | | | | |
|  |  |  |  |  |  |  |  |  |  |  |  |  |  |  |  |  |  |  |  |  |  |  |  |  | | | | | | | | | | | | |
|  |  |  |  |  |  |  |  |  |  |  |  |  |  |  |  |  |  |  |  |  |  |  |  |  | | | | | | | | | | | | |
|  |  |  |  |  |  |  |  |  |  |  |  |  |  |  |  |  |  |  |  |  |  |  |  |  | | | | | | | | | | | | |
|  |  |  |  |  |  |  |  |  |  |  |  |  |  |  |  |  |  |  |  |  |  |  |  |  | | | | | | | | | | | | |
| 1. **VOD after ACT** | | | | | | | | | | | | | | | | | | | | | | | | | | | | | | | | | | | | |
| Diagnosis date | | | | | | Treatment | | | | | | | | | | | | | | | | | | | | | | | | | | | | | | |
|  | | | | | | Date | | | | | | | | | | | | Administration drug | | | | | | | | | | | | | | | | | | |
|  |  |  |  |  |  |  | | | | | | | | | | | |  | | | | | | | | | | | | | | | | | | |
|  |  |  |  |  |  |  | | | | | | | | | | | |  | | | | | | | | | | | | | | | | | | |
|  |  |  |  |  |  |  | | | | | | | | | | | |  | | | | | | | | | | | | | | | | | | |
|  |  |  |  |  |  |  | | | | | | | | | | | |  | | | | | | | | | | | | | | | | | | |
|  | | | | | |  | | | | | | | | | | | |  | | | | | | | | | | | | | | | | | | |
|  |  |  |  |  |  |  | | | | | | | | | | | |  | | | | | | | | | | | | | | | | | | |
|  |  |  |  |  |  |  | | | | | | | | | | | |  | | | | | | | | | | | | | | | | | | |
|  |  |  |  |  |  |  | | | | | | | | | | | |  | | | | | | | | | | | | | | | | | | |
|  |  |  |  |  |  |  | | | | | | | | | | | |  | | | | | | | | | | | | | | | | | | |
| 1. **Completion of Study Drug** | | | | | | | | | | | | | | | | | | | | | | | | | | | | | | | | | | | | |
| Completion | | | | | | □ completion □ incompletion (switch to other drugs) | | | | | | | | | | | | | | | | | | | | | | | | | | | | | | |
| Cause of  incompletion | | | | | | □ Adverse effect □ Compliance □ Mucositis progression  □ GVHD □ etc. ( ) | | | | | | | | | | | | | | | | | | | | | | | | | | | | | | |
| Switch of study drug | | | | | | Date | | | | | | | | | | | | Administration drug | | | | | | | | | | | | | | | | | | |
|  |  |  |  |  |  |  | | | | | | | | | | | |  | | | | | | | | | | | | | | | | | | |
|  |  |  |  |  |  |  | | | | | | | | | | | |  | | | | | | | | | | | | | | | | | | |
|  |  |  |  |  |  |  | | | | | | | | | | | |  | | | | | | | | | | | | | | | | | | |
|  |  |  |  |  |  |  | | | | | | | | | | | |  | | | | | | | | | | | | | | | | | | |
|  |  |  |  |  |  |  | | | | | | | | | | | |  | | | | | | | | | | | | | | | | | | |

별첨10. 피험자 보상에 대한 규약

**피험자 보상에 대한 규약**

본 피험자 보상에 대한 규약은 서울대학교 의과대학 혈액종양내과 교수 윤성수(이하 “연구챔임자”)가 의뢰하는 임상시험에 참여하여 임상시험용의약품을 투여 받은 피험자(이하 “피험자”)에 대하여 적용되는 보상규약입니다.

1. **보상원칙**

연구책임자는 의약품임상시험관리기준에 따라 이루어진 임상시험에 있어서 임상시험용 의약품 또는 임상시험 절차로 인하여 피험자에게 발생한 유해하고 의도되지 않은 반응에 의한 신체상의 손상에 대하여 본 보상규약 및 관련법률의 규정에 따라 이를 보상합니다.

1. 일시적인 통증 또는 쉽게 치료될 수 있는 정도의 손상으로서 연구책임자 및 의료진이 이에 대한 적절한 치료가 필요하다고 판단하는 경우(보상의 범위는 필요한 치료비에 한정함); 또는
2. 입원 또는 입원기간의 연장이 필요한 경우; 또는
3. 지속적 또는 의미 있는 불구나 기능저하를 초래하는 경우; 또는
4. 선천적 기형 또는 이상을 초래하는 경우; 또는
5. 사망을 초래하거나 생명을 위협하는 경우
6. **보상 요건**

본 보상규약에 따른 피험자 보상은 다음의 요건하에 이루어 집니다.

1. 본 임상시험용의약품 또는 임상시험 절차로 인하여 발생한 신체상의 손상일 것
2. 연구담당자가 식품의약품안전청장 혹은 임상시험심사위원회의 승인을 받은 임상계획서의 제반 내용을 준수하였을 것
3. 피험자가 연구책임자 또는 연구담당자의 제반 지시사항을 모두 준수하였을 것
4. **보상제외 사유**

전 2조의 규정에 불구하고 다음의 경우에는 보상에서 제외 됩니다.

1. 연구책임자가 의뢰하지 않았거나, 제공하지 않은 임상시험용 의약품으로 인해 발생한 피해
2. 임상시험용의약품으로부터 기대된 효과 또는 혜택을 제공하지 못한 데에 대한 보상 (피험자의 기왕증의 진행 및 악화로 인한 경우 포함).
3. 피험자의 부주의로 인하여 발생한 피해
4. 임상시험용의약품 및 임상시험 절차와 관련성이 없는 경우
5. 임상시험 종료 후 시험약의 계속 투여에 의한 경우
6. **보상 기준**
7. 임상시험 기간 도중 피험자에게 발생한 피해가 시험약 또는 임상시험 절차와 관련이 없음을 입증하지 못하는 경우 연구책임자는 의료보험이나 다른 방법으로 보상되지 않는 부분에 대해 합리적인 치료비를 보상합니다.
8. 보상 수준은 피해의 본질, 그 정도, 지속성 여부, 유사사례 등을 종합적으로 고려하여 당사자들간의 합의한 보상방법에 따라 보상합니다.
9. 피험자와 연구책임자 사이에 합의가 이루어지지 아니한 경우에는, 법원의 판결 및 이에 준하는 결정의 확정내용에 따라 보상합니다.
10. **보상 절차**
11. 본 보상규약에 따른 신체상의 손상을 입은 피험자는 임상시험의 연구책임자나 시험기관에 먼저 필요한 의료조치를 요청하여야 합니다.
12. 연구책임자나 시험기관의 조치에도 불구하고 신체상의 손상이 완치되지 아니한 피험자는 연구책임자에 대하여 이에 대한 보상을 요청할 수 있습니다.
13. 연구책임자는 위 보상요청을 받은 후 지체 없이 보상대상 해당여부 및 보상기준에 대한 조사를 마치고 이에 관한 내용을 피험자에게 통보합니다.
14. 피험자는 위 통보내용에 대하여 이의가 있는 경우, 위 통보를 받은 날로부터 30일 이내에 이에 대한 이의내용을 연구책임자에게 통보하여야 합니다.
15. 피험자가 제(3)항의 통보를 받고도 이에 대한 이의를 통보하지 아니한 경우, 양 당사자는 위 통보내용에 따른 보상에 합의한 것으로 양해합니다.
16. 피험자가 제(4)항의 규정에 따라 이의를 통보한 경우, 양자가 수용할 수 있는 전문가의 자문을 통해 보상수준을 결정하고 이에 따라 보상합니다.
17. **적용범위**
18. 본 보상규약은 연구책임자가 의뢰하는 모든 임상시험에 참여하는 피험자에 대하여 연구책임자와 피험자간에 다른 약정이 없는 한 그 범위 내에서 일반적으로 적용됩니다.
19. 피험자가 임상시험에 대한 보상에 관하여 연구책임자의 승인을 받지 아니하고 임상시험과 관련된 다른 제3자와 체결한 일체의 합의내용은 연구책임자에 대하여 효력이 없습니다.

연구책임자: 서울대학교 의과대학 혈액종양내과

교수 윤성수 (인)
