# Supplementary material for: Topical Recombinant Human Epidermal Growth Factor for Oral Mucositis Induced by Intensive Chemotherapy with Hematopoietic Stem Cell Transplantation: Final Analysis of a Randomized, Double-Blind, Placebo-Controlled, Phase 2 Trial
Source: PLoS One. 2017 Jan 3;12(1):e0168854. doi: 10.1371/journal.pone.0168854 (PMC5207736; doi:10.1371/journal.pone.0168854)
Supplement: S2 Protocol — (DOCX) [file pone.0168854.s005.docx]

**Protocol Summary in English**

**Title:** Randomised phase II study of recombinant human epidermal growth factor (rhEGF) on oral mucositis induced by intensive chemotherapy for hematologic malignancies

**ClinicalTrials.gov identifier:** NCT00845819

**1. Introduction**

Oral mucositis (OM) is one of the most common adverse events during chemotherapy and affects quality of life of patients receiving chemotherapy in relation to the dose of drugs. However, there is only one drug (palifermin) approved by the US FDA for the prevention of OM and the other methods to prevent or treat OM are just empirical and lack evidences. The results of recent study demonstrated promising efficacy and minimal toxicity of recombinant human epidermal growth factor (rhEGF) as a preventive drug of OM in head and neck cancer patients undergoing radiotherapy (Wu HG, et al. Cancer 2009;115(16):3699-3708). This clinical trial is a double-blind, randomised, prospective, single-institutional, phase II study to evaluate efficacy and toxicity of rhEGF as a preventive drug of OM during intensive chemotherapy with stem cell transplantation in patients with hematologic malignancies.

**2. Study eligibility criteria**

1. Inclusion criteria

- Patients with more than 18 years of age
- Patients with confirmed diagnosis of hematologic malignancies including acute & chronic leukemia, lymphoma, plasma cell dyscrasia, myelodysplastic syndrome, aplastic anemia, etc.
- Patients who are planned to receive high-dose chemotherapy with hematopoietic stem cell transplantation
- ECOG performance status of 0-2
- Informed consent

1. Exclusion criteria

- Patients having previous history of hypersensitivity to this drug or similar drugs
- Patients having oral ulcer or herpes at the time of inclusion
- Patients having severe dental disease at the time of inclusion
- Patients received chemotherapy or radiotherapy within 3 weeks
- Patients received surgery within 3 weeks
- Patients who had finished clinical trials which could affect the results of this trial within 4 weeks or are attending one at the time of inclusion
- Patients having another diseases which have worse prognosis than patients' hematologic malignancy
- Patients with major psychotic disorder or drug/alcohol abuser
- Women who are pregnant or breastfeeding
- Inappropriate patients according to the investigators' opinion
- Refusal at patients' will

**3. Study endpoints**

1. Primary endpoint

- Incidence of OM of grade 2 or higher (NCI CTCAE [common terminology criteria for adverse events] 3.0)

1. Secondary endpoints

- Day of onset and duration of OM of grade 2 or higher (NCI CTCAE 3.0)
- Incidence, day of onset, and duration of OM of grade 3 or higher (NCI CTCAE 3.0)
- Incidence, day of onset, and duration of OM of grade 2 or higher (WHO OM scoring system scale)
- Incidence, day of onset, and duration of OM of grade 3 or higher (WHO OM scoring system scale)
- Incidence, day of onset, and duration of OM of grade 4 or higher (WHO OM scoring system scale)
- OM daily questionnaire (OMDQ) score during treatment
- Duration of opioid analgesics use
- Side effects and safety evaluation of rhEGF
- Adverse events

**4. Statistics (including power calculations)**

1. Study design

Double blind, placebo-controlled, randomised method, single-centre study


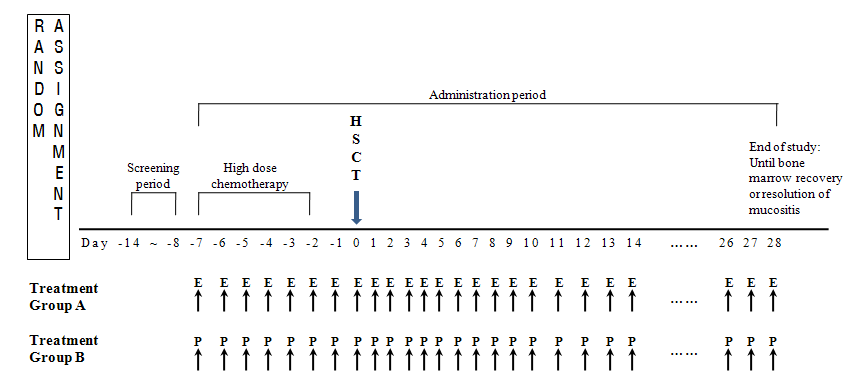


Figure 1. Study design and schedule. HSCT, Hematopoietic Stem Cell Transplantation; Treatment Group A, recombinant human epidermal growth factor (E); Treatment Group B, placebo (P).

1. Sample size calculation

This study is designed to test the hypothesis that the incidence of NCI grade ≥2 OM would be 37% (H1), which is significantly different from 64% of previously published data. This study is designed to have 80% power to detect 27% improvement in the incidence of NCI grade ≥2 OM in the rhEGF group as compared with placebo, at a two-sided alpha level of 0.05. If patients are randomised at 1:1 ratio, to reject H0, at least 62 patients are required in each treatment group. Assuming a 10% dropout rate, the total number of patients needed for this study is 138 patients - 69 patients per each group.

| **Numeric Results for Two-Sided Test of Proportions. Continuity Correction Applied.**  **Power N1 N2 Alpha Beta P1 P2**  0.806915 62 62 0.050000 0.193085 0.37 0.64 |
| --- |

1. Randomisation

- Patients will be randomly assigned to the rhEGF or placebo group at a 1:1 ratio using computer-generated randomisation by the Medical Research Collaborating Center (MRCC), Seoul National University Hospital (Seoul, Republic of Korea), which is not involved in the data analysis. The study is double blinded, thus all clinicians, patients, and investigators assessing outcomes, and analysing data are masked to treatment assignment. The placebo is compounded with all the ingredients of the rhEGF preparation except for rhEGF, and is supplied in a masked manner for patients.

1. Administration of study drugs

- Dose of investigational products
  - Patients are instructed to spray rhEGF or placebo over their entire oral mucosa twice daily.
- Methods of administration
  - For each application, patients spray total 6 times at palate, oropharynx, both buccal mucosa, tongue, and gingiva. Oral intake is restricted 30 minutes after administration.
- Duration of administration
  - Application of drugs begins with start of intensive chemotherapy and ends at the time of recovery from neutropenia (ANC>1000/µL for 3 days) or disappearance of oral mucositis.

1. Clinical data collection

- Assessment of OM severity
  - OM severity is assessed once during screening period and every day during administration period.
  - A qualified researcher grades the severity of OM using the following two scales: the NCI CTCAE ver. 3.0 and the WHO oral mucositis scoring system scale.
- Self-assessment of mouth and throat soreness and swallowing difficulty
  - Patient-reported quality of life during the study period is evaluated daily using a modified version of the Oral Mucositis Daily Questionnaire (OMDQ).
- Medical records
  - Concomitant medications including total parenteral nutrition (TPN), opioid analgesics, and antibiotics are recorded in the case report form every day.
  - In case of infection, causative organisms, severity, duration, and treatment methods are recorded in the case report form.
  - Transplant-related complications such as acute graft-versus-host disease and veno-occlusive disease are recorded in the case report form along with their severity, duration, and treatment methods.
- Advese events
  - Treatment-related adverse events are monitored every day.
  - In case of adverse event development, related information such as date of onset, severity, relationship with investigational products, and treatment methods is recorded and evaluated.
  - Serious adverse events are reported to clinical trial review board.
- Compliance
  - Patient compliance is assessed through daily confirmation of patient-documented administration records.
  - (Compliance) = (Actual administration by patient) / (Scheduled administration)

1. Principles of data analysis

The final data will be analysed on an intent-to-treat (ITT) basis for primary and secondary endpoints. In addition, per protocol analysis will be performed for patients whose compliance for study drugs is more than 75%. The t-test and Mann-Whitney U test will be used to compare parametric and non-parametric continuous variables, respectively. Categorical variables will be compared using the Pearson’s chi-square test or the Fisher’s exact test, as appropriate.

1. Interim analysis

Interim analysis is planned at the time of study design using the O’Brien-Fleming type alpha spending function by Lan and Demets (1983). If the interim analysis is performed when 50% of patients were enrolled, an alpha level of 0.003 should be used to reject H0.

| **Details when Spending = O'Brien-Fleming, N1 = 62, N2 =62, P1 = 0.37, P2 = 0.64,**  **Continuity Correction.**  **Lower Upper Nominal Inc Total Inc Total**  **Look Time Bndry Bndry Alpha Alpha Alpha Power Power**  1 0.50 -2.96259 2.96259 0.003051 0.003051 0.003051 0.168499 0.168499  2 1.00 -1.96857 1.96857 0.049002 0.046949 0.050000 0.638415 0.806915 |
| --- |
